# Multivariate genome-wide analysis reveals shared genetic architecture and brain structural correlates of human cognitive abilities

**DOI:** 10.1038/s41598-025-25509-z

**Published:** 2025-11-24

**Authors:** Haifeng Chen, Yuxiong Liao, Luejun Tang, Xiaoyun Wei, Tongshun Li, Wei Chen

**Affiliations:** Department of Brain Diseases, Ward 1, Nanning Hospital of Traditional Chinese Medicine, Affiliated to Guangxi University of Chinese Medicine, No. 45 Beihu North Road, Xixiangtang District, Nanning, 530000 China

**Keywords:** Cognitive abilities, Genome-wide association study, Genomic structural equation modeling, Transcriptome-wide association study, Brain imaging genetics, Polygenic risk score, Fine-mapping, Neurogenetics, Computational biology and bioinformatics, Genetics, Neuroscience

## Abstract

Limited knowledge currently exists regarding the shared genetic architecture influencing human cognitive ability-related traits. We utilized Genomic Structural Equation Modeling (Genomic-SEM) along with multiple post-GWAS methods to estimate potentially causal single nucleotide polymorphisms (SNPs) associated with cognitive ability phenotypic variation. Our study identified 3,842 genome-wide significant loci, including 275 novel loci. Applying multiple transcriptome-wide association methods, we analyzed susceptibility gene signal loci highly correlated with cognitive ability GWAS from tissue, cellular, and genomic element perspectives, identifying 13 high-confidence candidate causal genes and related functional element information. Subsequently, we systematically evaluated 80–90% of currently known human common disease data in the IEU database, along with 3,935 brain imaging-derived phenotypes from UK Biobank and cortical morphological features from 51,665 individuals in the ENIGMA database to determine cognitive ability-related susceptibility factors. We applied the BrainXcan pipeline for brain imaging genetic analysis of cognitive abilities. Additionally, we used summary data-based polygenic scoring methods to systematically analyze the genetic contribution of different chromosomes to cognitive abilities and their risk prediction value. Our study, through multivariate GWAS analysis of cognitive ability common genetic factors, contributes to mapping the shared genetic architecture of human cognitive abilities.

**Supplementary Information:**

The online version contains supplementary material available at 10.1038/s41598-025-25509-z.

## Introduction

As Einstein once noted, “Imagination is more important than knowledge.” Cognitive abilities are not merely numbers on intelligence tests, but rather complex and dynamic neurobiological processes characterized by fine-tuned neural networks and synaptic plasticity changes that are profoundly influenced by genetic, environmental, and lifestyle factors^1^. With increasing global demands for human capital and educational quality, individual differences in cognitive abilities have an increasingly significant impact on academic achievement, career development, and quality of life, making them important concerns in education, medicine, and socioeconomic fields^2^. Despite significant advances in neurocognitive science and educational neuroscience in recent years, our understanding of the specific genetic and biological mechanisms underlying cognitive abilities remains limited^3^.

Research suggests that differences in neurotransmitter system function and synaptic plasticity changes are considered important biological foundations for individual differences in cognitive abilities^4,5^. However, these findings are still insufficient to fully explain the vast differences in cognitive performance and learning abilities among individuals. Twin and family studies have consistently demonstrated that cognitive abilities are substantially heritable, with heritability estimates ranging from 50 to 80% across the lifespan^6,7^. Yet despite this strong evidence for genetic influence, genome-wide association studies (GWAS) have historically struggled to identify the specific genetic variants responsible for this heritability, a phenomenon known as the “missing heritability” problem^8,9^.

To address these challenges, this study aims to dissect the underlying molecular mechanisms of cognitive abilities through the integration of multiple genetic analysis tools and strong association exploration methods, while exploring associations with various neuropsychiatric disorders. In particular, we focus on multiple genomic loci and chromosomal regions related to cognitive abilities to reveal potential intervention targets for promoting cognitive function and preventing cognitive decline^10^. This research not only expands our understanding of the genetic foundations of human cognitive abilities but also provides theoretical and practical support for intervention strategies addressing global educational equity and cognitive health challenges.

To address the current lack of systematic measurement of the shared genetic architecture of cognitive abilities, we designed a multivariate GWAS study targeting latent multidimensional cognitive ability common factors. We employed genomic structural equation modeling (Genomic SEM)^11^, applying it to published GWAS summary statistics related to intelligence^12^, educational attainment^13^, processing speed^14^, executive function^15^, memory performance^16^, and reaction time. By integrating these statistics, we obtained SNP association effects on latent cognitive ability common factors, thereby constructing a GWAS study targeting multidimensional cognitive ability phenotypes that have never been directly measured.

We further adopted comprehensive analysis methods from systems biology, defining the portion of genetic variation in multidimensional cognitive abilities that is not explained by single cognitive domains as the shared genetic foundation of cognitive abilities, and conducted multiple post-GWAS analyses on this foundation^17,18^. Although this approach is not perfect for the true relationship between cognitive ability-related neural pathways and multifactorial interactions, since cognitive ability is a complex process driven jointly by genetic, environmental, and stochastic factors, this analysis excludes confounding effects based on single cognitive indicators, thereby enabling systematic analysis of the previously difficult-to-study common genetic architecture of cognitive abilities^19,20^.

Finally, we validated the neurobiological foundations of cognitive abilities through the BrainXcan pipeline and brain imaging phenotype analysis, and conducted large-scale association analysis with 50,033 human phenotypes^21^. Our aim is to construct an easily understandable genetic risk map of cognitive abilities for clinicians, educators, and others, enabling them to directly apply genetic information to develop individualized cognitive training and intervention measures. Our research aims to establish a complete translational pathway from genomic statistics to clinical educational practice.

## Methods

**Fig. 1:**
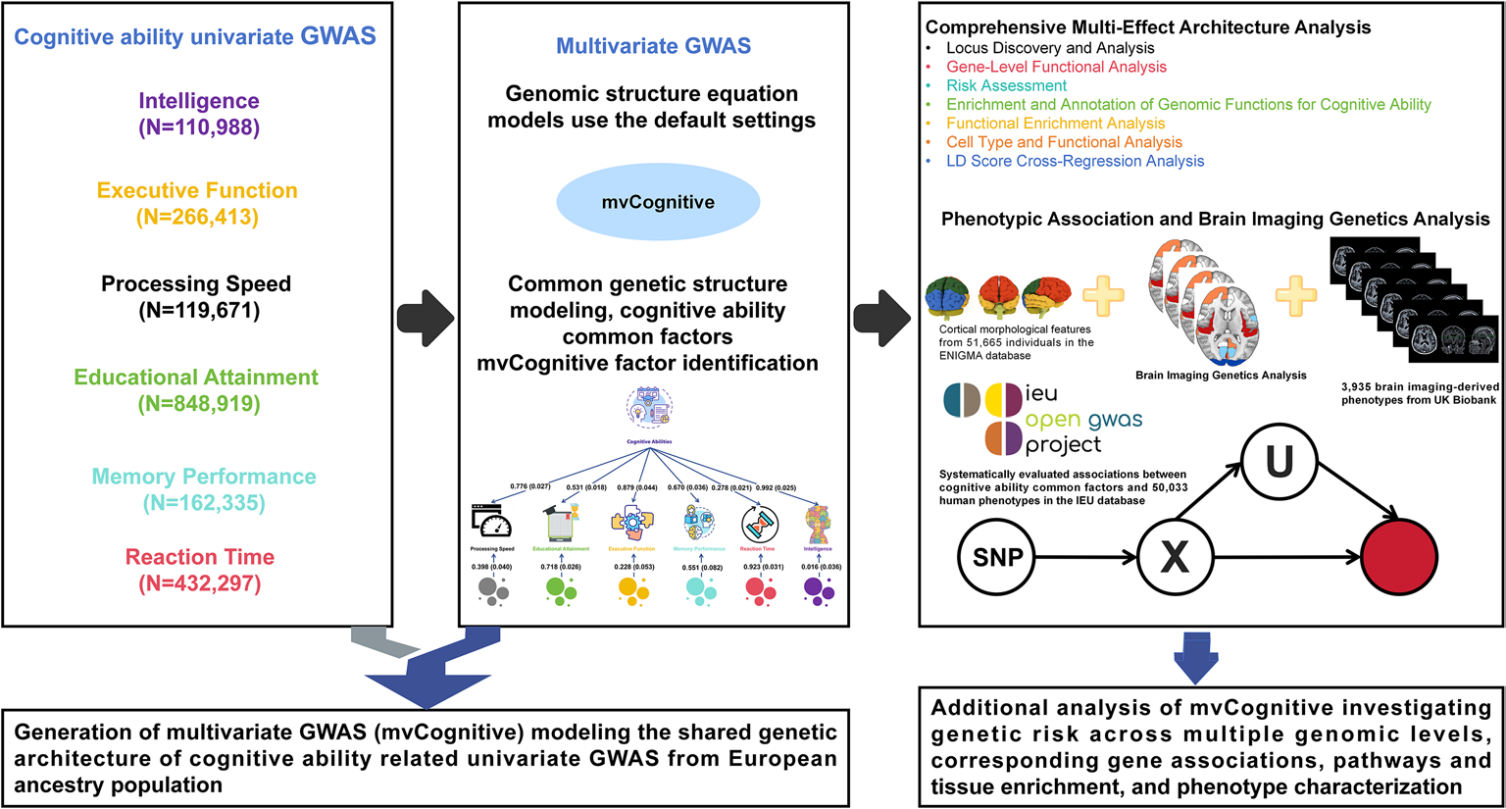
Study design and analytical workflow for multivariate genetic analysis of cognitive abilities. (a) Input data from six cognitive-related univariate GWAS including Intelligence (n = 110,988), Executive Function (n = 266,413), Processing Speed (n = 119,671), Educational Attainment (n = 848,919), Memory Performance (n = 162,335), and Reaction Time (n = 432,297). (b) Genomic structural equation modeling (Genomic SEM) to identify a common cognitive factor (mvCognitive) with factor loadings shown for each phenotype. The model demonstrates shared genetic architecture across cognitive domains using default settings for common genetic structure modeling. (c) Comprehensive downstream analyses including locus discovery, gene-level functional analysis, risk assessment, enrichment analysis, cell-type analysis, and LD Score regression analysis. (d) Phenotypic association analysis using 50,033 human phenotypes from the IEU database and brain imaging genetics analysis using cortical morphological features from 51,665 individuals in the ENIGMA database and 3,935 brain imaging-derived phenotypes from UK Biobank. The workflow generates a multivariate GWAS (mvCognitive) modeling the shared genetic architecture of cognitive abilities and enables investigation of genetic risk across multiple genomic levels, corresponding gene associations, pathways, tissue enrichment, and phenotype characterization. Total effective sample size represents 1,219,586 SNPs from European ancestry populations.

A flowchart overview is presented in Fig. 1.

### GWAS input data sources

The input GWAS data for our cognitive ability structural equation modeling came from six cognitive-related trait GWAS, including Intelligence, Executive Function, Processing Speed, Educational Attainment, Memory Performance, and Reaction Time. All input GWAS studies had ethical approval from their respective institutional review boards, and all participants provided informed consent, with data undergoing rigorous quality control^22^.

1. Intelligence GWAS data were obtained from Hu et al. 2025 (GCST90310159, n = 110,988)^23^. This study was based on UK Biobank fluid intelligence scores (data field 20,016), primarily from individuals of British ancestry, using standardized intelligence assessment tools, providing an important foundation for genetic research on general cognitive ability. This dataset has high phenotypic measurement precision and serves as a core phenotype for cognitive ability research.
2. Executive Function GWAS data came from Perry et al. 2025 (GCST90503115, n = 266,413)^24^. This study analyzed multiple executive function GWAS data through genomic structural equation modeling, improving gene discovery efficiency and providing valuable genetic information for higher-order cognitive control and cognitive flexibility. Executive function, as a core component of cognitive abilities, has important clinical and educational significance.
3. Processing Speed GWAS data were from Carey et al. 2024 (GCST90309368, n = 119,671)^25^. This study used confirmatory factor analysis (factor 34) to comprehensively evaluate cognitive and processing speed phenotypes, providing an important foundation for genetic research on cognitive processing efficiency. Processing speed, as a fundamental dimension of cognitive ability, reflects the information transmission efficiency of the nervous system.
4. Educational Attainment GWAS came from Xia et al. 2025 (GCST90566697, n = 848,919)^26^. This study used multi-trait analysis and genomics (MTAG) methods to analyze educational attainment data, providing strong statistical power for understanding the genetic basis of educational achievement. Educational attainment, as an important external manifestation of cognitive ability, has the largest sample size advantage.
5. Memory Performance GWAS data were from Hatoum et al. 2022 (GCST90179116, n = 162,335)^27^. This study evaluated genetic variation in memory function based on prospective memory tasks, providing a foundation for research on genetic mechanisms of memory-related cognitive processes. Memory performance, as an important component of cognitive ability, reflects the capacity for information encoding, storage, and retrieval.
6. Reaction Time GWAS data came from Hatoum et al. 2022 (GCST90179115, n = 432,297)^14^. This study provided important statistical power for understanding the genetic basis of cognitive processing speed and neural conduction efficiency based on reaction time measurements. Reaction time, as a fundamental measurement indicator of cognitive ability, reflects the basic functional efficiency of the nervous system.

All analyses in this study were based on publicly available GWAS summary statistics rather than individual-level genotype data. The constituent GWAS had performed quality control procedures including sample quality screening, single nucleotide polymorphism (SNP) quality screening (MAF > 0.01, INFO > 0.8), population stratification correction, and relatedness adjustment^28^. To ensure data consistency, all analyses were based on the GRCh37/hg19 reference genome and used the 1000 Genomes Project European population as the LD reference panel^29^.

### Quality control of input GWAS

As our analysis utilized publicly available GWAS summary statistics, sample-level quality control and relatedness adjustment were performed by the original GWAS study teams prior to our analysis.

1. Sample-level quality control: Constituent GWAS studies applied standard procedures including exclusion of samples with low genotyping call rate (< 95%), sex discordance (between reported and genetic sex), abnormal heterozygosity (± 3 standard deviations from population mean), and non-European ancestry (determined by principal components analysis with reference populations).
2. Relatedness adjustment: Related individuals (kinship coefficient > 0.0884, approximately third-degree relatives or closer) were identified and either one individual per related pair was excluded, or association testing was performed using linear mixed models that account for genetic relatedness matrix (in large biobank studies).
3. MHC region handling: The major histocompatibility complex (MHC) region located on chromosome 6 (approximately 25,000,000–35,000,000 bp) received special treatment due to its genetic diversity and structural complexity, particularly the polymorphisms of immune-related genes^30^.
4. SNP-level filtering in our analysis: We included all autosomal SNPs from the six input GWAS after quality control screening. SNPs were filtered to the 1000 Genomes Phase 3 EUR panel, removing SNPs with MAF < 0.01 (due to few samples within genotype clusters, these SNPs are error-prone and typically have high LD score regression standard errors), removing SNPs with zero effect estimates (to avoid affecting matrix reactivity, which is necessary for genomic structural equations), removing SNPs that do not match the reference panel, and removing SNPs with allele mismatches^31^.

### Sample overlap assessment

In our analysis, the input GWAS came from different genomic information storage sites with distinct participants across studies. This means we adequately considered sample intersections between different cohorts during GWAS analysis to ensure result accuracy and generalizability, while also considering potential statistical impacts from sample overlap^32^.

Genomic SEM inherently accounts for sample overlap through its use of genetic covariance matrices derived from GWAS summary statistics rather than individual-level data, thereby avoiding biases that could arise from overlapping participants across different studies. The method’s reliance on LD Score regression for genetic covariance estimation provides robust results even in the presence of sample overlap.

### Genomic structural equation modeling

1. Rationale for Genomic Structural Equation Modeling. We selected Genomic SEM for its ability to explicitly model latent genetic factors underlying correlated phenotypes while accounting for sample overlap through LD Score regression. Unlike alternatives such as MTAG or genomic PCA, Genomic SEM provides interpretable theoretical constructs (the common cognitive factor) rather than atheoretical linear combinations, with formal model fit evaluation (CFI, SRMR, RMSEA) and SNP-level heterogeneity testing to identify domain- specific effects.
2. We implemented genomic structural equation modeling (Genomic SEM) using the GenomicSEM R package (v.0.0.5)^33^ to conduct genomic structural equation GWAS analysis of intelligence, executive function, processing speed, educational attainment, memory performance, and reaction time, investigating the broad common genetic foundation underlying these cognitive ability-related phenotypes. Genomic SEM is a newly developed multivariate method capable of studying multiple potential multivariate models and exploring the potential common genetic structure of the cognitive ability spectrum^34^.See Table 1 for details.
3. Genomic SEM is not affected by bias from sample overlap (e.g., potential overlap of participants in different cognitive research cohorts) or sample size imbalances^35^. It also facilitates identification of genetic variants that affect only some, not all, cognitive phenotypes, thus not representing broad cross-cognitive domain genetic associations but potentially reflecting cognitive domain-specific genetic mechanisms.
4. Genomic SEM proceeds in two stages. Stage 1 estimates the empirical genetic covariance matrix and corresponding sampling covariance matrix. We prepared summary statistics from cognitive ability-related trait GWAS for Stage 1 and used multivariate extensions of cross-phenotype LD score regression to generate empirical genetic covariance matrices among the six cognitive phenotypes as input for the SEM common factor model^36^. Stage 2 specifies an SEM model that minimizes differences between the hypothesized covariance structure and the empirical covariance matrix calculated in Stage 1. Here, our main research objective was to identify the common genetic architecture underlying the six cognitive ability-related phenotypes; therefore, we tested a single-factor model to characterize the cognitive ability common factor. Model fit was evaluated using SRMR, model χ^2^, Akaike Information Criterion (AIC), Comparative Fit Index (CFI), and Root Mean Square Error of Approximation (RMSEA)^37^. By applying appropriate common factor SEM specifications, individual autosomal SNP associations were incorporated into genetic and corresponding sample covariance matrices, generating cognitive ability common factor structural equation genome-wide association analysis results for approximately 1,219,586 SNPs sharing covariance among the six input cognitive ability-related GWAS.

**Table 1:** Parameters and quality metrics for genomic structural equation modeling.

Phenotype	NSNPs	h2_se	λGC	Mean_ChiSquare	Intercept_se	Ratio_se
Processing speed	1169,915	0.1171 (0.0067)	1.2059	1.2662	0.9893 (0.0083)	-0.0401 (0.031)
Executive function	490,014	0.1214 (0.0061)	1.548	1.7391	1.0064 (0.0188)	0.0087 (0.0254)
Educational attainment	1132,654	0.1085 (0.0032)	2.0986	2.7607	0.9213 (0.0158)	-0.0447 (0.009)
Memory performance	1122,102	0.0364 (0.0035)	1.0966	1.1251	1.0068 (0.0075)	0.0543 (0.0596)
Reaction time	1122,218	0.0832 (0.0026)	1.557	1.7497	1.0347 (0.0113)	0.0462 (0.0151)
Intelligence	1180,233	0.2335 (0.0103)	1.4256	1.5419	1.0289 (0.0099)	0.0533 (0.0183)

*N SNPs* Number of single nucleotide polymorphisms included in analysis, *λGC* genomic control inflation factor, *χ*^*2*^ chi-square statistic; Intercept and Ratio refer to LD Score regression intercept and ratio parameters.

### SNP heterogeneity assessment

To assess whether SNP associations in our cognitive ability structural equation GWAS were appropriately modeled within the multivariate structural equation model (SEM) framework, we calculated SNP heterogeneity statistics (Q_SNP)^38^. The null hypothesis of this test is that SNP associations in individual phenotype GWAS can be completely statistically mediated through the cognitive ability structural equation model. Therefore, significant Q_SNP values (*P* < 0.05) in our cognitive ability structural equation GWAS indicate that specific SNPs exert effects through pathways other than the shared genetic mechanisms established for cognitive ability-related diseases in the model.Importantly, SNPs with significant Q_SNP values were retained in our analysis rather than excluded. Unlike traditional fixed-effects meta-analysis where heterogeneous SNPs are typically removed, the Genomic SEM framework interprets Q_SNP heterogeneity as biologically informative, representing domain-specific genetic effects that diverge from the common factor pathway. Excluding such SNPs would discard variants with potential specialized roles in specific cognitive domains.

### Multi-level model assessment and locus definition

We conducted multi-level strategic adjustments to our genomic structural equation model, including setting different significance thresholds (*P* < 5 × 10^–16^ and *P* < 5 × 10^–12^) to identify novel SNP loci at different confidence levels, balancing statistical power with false positive control^39^. We used FUMA (Functional Mapping and Annotation of Genetic Associations) to identify genomic loci and find lead SNPs associated with our cognitive ability structural equation GWAS that have low linkage disequilibrium (LD < 0.1) with other SNPs and genome-wide significance (*P* < 5 × 10^–8^)^40^. Novel loci were defined as those > 1 Mb distant from previously identified loci in univariate GWAS data. LD clumping was performed using a window size of 250 kb and r^2^ < 0.1 threshold to define independent lead SNPs, with FUMA automatically performing conditional analysis within each locus to identify independent secondary signals.

### Fine-mapping analysis

To identify the most likely causal variants associated with our cognitive ability structural equation GWAS, we used SuSIE and FINEMAP, implemented in the echolocatoR R package v.2.0.3^41,42^. We set a probability threshold of 0.95 to define credible sets of potentially causal variants. We used 250 kb windows around each lead SNP to calculate causal inference probabilities for each SNP within these regions. The echolocatoR defines a ‘consensus SNP’ as variants appearing in both SuSIE and FINEMAP results, with average posterior probabilities calculated for these consensus SNPs.

### Transcriptome-wide association studies (TWAS)

Following identification of potentially causal variants, we conducted TWAS to prioritize genes associated with our cognitive ability structural equation GWAS based on relationships between gene expression and phenotypes^43^. We used the FUSION method for TWAS with pre-computed expression quantitative trait loci (eQTL) features from GTEx v.8 data (37,920 gene/tissue pairs)^44^.

We applied a two-tier significance threshold strategy: (1) Bonferroni correction for 37,920 gene-tissue pairs tested (*P* < 1.32 × 10^–6^) to identify high-confidence transcriptome-wide significant genes, and (2) nominal significance (*P* < 0.05) to retain genes for comprehensive pathway enrichment and cross-method validation analyses, following established practices in GWAS gene-set analysis.

For TWAS-significant genes, we further applied FOCUS (Fine mapping Of Causal gene Sets), a fine-mapping method specifically designed for TWAS studies^45^. FOCUS evaluates whether genes have causal relationships with phenotypes based on posterior inclusion probabilities. We considered TWAS-significant genes that showed consistency with other evidence as potentially causal^46^.

### Functional enrichment analysis

We used MAGMA and FUMA (GESA) for gene enrichment analysis and gene pathway set analysis, investigating potential relationships between our cognitive ability structural equation GWAS and Mendelian disease genes and their related pathways^47^. Additionally, we used MendelVar (https://mendelvar.mrcieu.ac.uk/submit/) for gene enrichment analysis^48^. For disease and phenotype enrichment analyses, empirical P-values from permutation testing were corrected for multiple testing using the Benjamini–Hochberg false discovery rate (FDR) method. Associations with FDR < 0.05 were considered statistically significant.

### Cell-type annotation analysis

To identify etiological cell types associated with our cognitive ability structural equation GWAS, we used Cell-type Expression-specific integration for Complex Traits (CELLECT) with single-cell RNA sequencing data^49^. We used the Tabula Muris dataset containing transcriptome data from 100,000 cells across 20 organs and tissues from mice. We preprocessed and normalized single-cell RNA sequencing data using CELLEX, calculating expression specificity scores for each gene^50^. Cell-type-specific analysis was performed using LDSC software with a false discovery rate (FDR) threshold of 0.05^51^.

### Partitioned heritability analysis

We used LDSC to calculate partitioned heritability of genomic regions^52^. By allocating phenotypic genetic information to different genomic regions (genes, enhancers, suppressors, etc.), we evaluated the contribution of each genomic region to phenotypic heritability. LDSC uses weighted LD matrices, genotype frequency files, and summary statistics to calculate and estimate genetic contributions of each region.

### Phenotypic association and brain imaging genetics analysis

To comprehensively assess associations between cognitive ability common factors and human diseases as well as neurobiological foundations, we conducted multi-level association analyses^53^. First, we systematically evaluated associations between cognitive ability common factors and 50,033 human phenotypes in the IEU database, covering 80–90% of currently known common human diseases, biomarkers, drug responses, and lifestyle factors^54^. Second, we used the BrainXcan pipeline for brain imaging genetic analysis of cognitive abilities to identify brain tissue-specific gene expression patterns related to cognitive abilities^55^. Additionally, we used Mendelian randomization methods to assess relationships between cognitive abilities and 3,935 brain imaging-derived phenotypes from UK Biobank, as well as cortical morphological features from 51,665 individuals in the ENIGMA database^56^. MR analyses employed multiple methods to assess robustness and detect horizontal pleiotropy: inverse variance weighted (IVW) as the primary method, supplemented by MR-Egger (testing directional pleiotropy), weighted median (robust to 50% invalid instruments), and weighted mode. Pleiotropy was evaluated using MR-Egger intercept test and Cochran’s Q statistic, with associations showing consistent directionality across methods and no significant pleiotropy (intercept *P* > 0.05) considered robust. Given the polygenic nature of the cognitive factor, we interpret results as associations with genetic predisposition rather than definitive causal effects.

### Polygenic risk score construction

We calculated polygenic risk scores (PRS) based on genome-wide summary statistics and assessed genetic contributions of different chromosomal regions to disease onset^57^. The specific method utilized PRS-CS (Polygenic Risk Score with Continuous Shrinkage) software through GWAS data and external LD reference panels to estimate posterior effect values of SNPs and calculate PRS^58^. This method employs Bayesian regression models, building on GWAS summary statistics and integrating LD reference panels to estimate effect values and calculate PRS.

## Results

### Structural equation model construction and statistical indicators

**Fig. 2:**
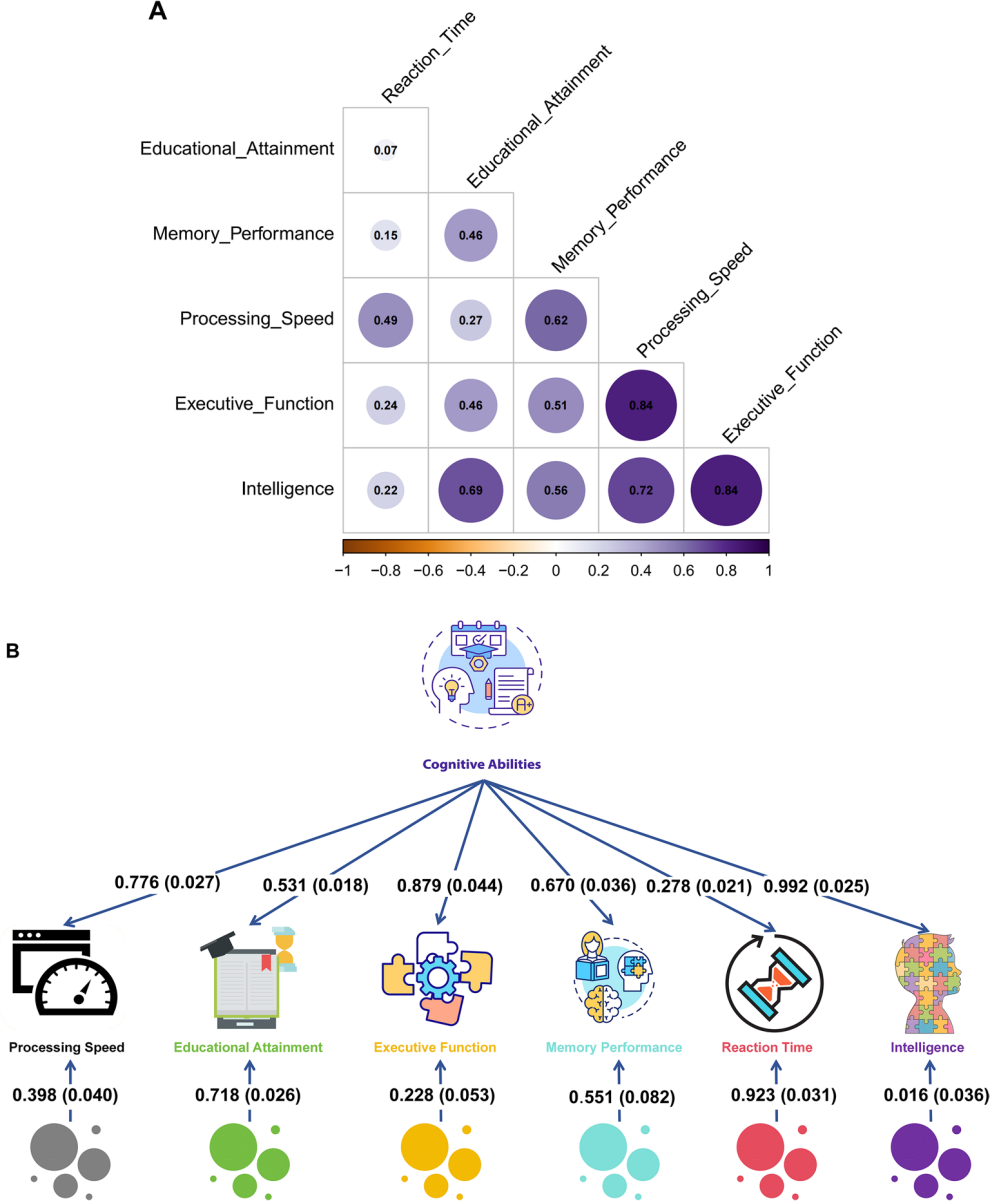
Genetic correlations and genomic structural equation modeling of cognitive abilities. (**A**) Genetic correlation matrix among six cognitive-related phenotypes based on LDSC analysis. Heat map colors represent the strength of genetic correlations, with darker colors indicating stronger positive correlations. Circle size represents the magnitude (absolute value) of genetic correlation. Diagonal elements (self-correlations, which equal 1.0 by definition) are excluded from visualization for clarity. All 15 unique pairwise genetic correlations were statistically significant (*P* < 0.0033 after Bonferroni correction for 15 tests), with correlation coefficients ranging from 0.07 (Reaction Time—Educational Attainment) to 0.84 (Processing Speed—Executive Function), supporting a shared genetic architecture across cognitive domains. (**B**) Genomic structural equation model (Genomic SEM) revealing a single common cognitive factor. Factor loadings (standard errors) for each cognitive phenotype onto the latent cognitive abilities factor are shown: Intelligence 0.992 (0.025), Executive Function 0.879 (0.044), Processing Speed 0.776 (0.027), Memory Performance 0.670 (0.036), Educational Attainment 0.531 (0.018), and Reaction Time 0.278 (0.021). Factor loadings represent standardized regression coefficients and are not constrained to sum to 1.0. Specific factor loadings representing phenotype-specific genetic variance not captured by the common factor are displayed below each phenotype. The model demonstrates that Intelligence shows the highest loading on the common cognitive factor, while Reaction Time retains the largest specific component. Model fit statistics indicate acceptable representation of the genetic covariance structure across the six cognitive phenotypes (CFI = 0.898, SRMR = 0.097).

LD Score regression analysis revealed the heritability contributions of the six univariate input GWAS as follows: Intelligence (h^2^ = 0.2335 (0.0103), Z = 22.7), Executive Function (h^2^ = 0.1214 (0.0061), Z = 19.8), Processing Speed (h^2^ = 0.1171 (0.0067), Z = 17.4), Educational Attainment (h^2^ = 0.1085 (0.0032), Z = 33.9), Reaction Time (h^2^ = 0.0832 (0.0026), Z = 31.8), and Memory Performance (h^2^ = 0.0364 (0.0035), Z = 10.4)^59^. Detailed single-factor genetic parameters are provided in Supplementary Tables 1 and 2, Fig. 2.

Prior to modeling, we conducted structural equation model analysis. The common factor model based on the genetic covariance matrix and empirical covariance matrix of the six input GWAS demonstrated acceptable fit (Comparative Fit Index (CFI) = 0.898, Standardized Root Mean Square Residual (SRMR) = 0.097)^60^. For detailed model stability assessment, see Supplementary Table 4a; for latent factor (F1) and univariate structural equation model parameters, see Supplementary Table 4b.

Within the single-factor model, Intelligence exhibited the highest factor loading (0.992), followed by Executive Function (0.879) and Processing Speed (0.776), while Educational Attainment (0.531), Memory Performance (0.670), and Reaction Time (0.278) showed moderate to lower loadings. Exploratory factor model analysis (reference Supplementary File 3) provided strong evidence for the existence of a shared genetic factor underlying cognitive abilities^33^.

While the high intelligence loading (0.992) reflects its strong relationship with general cognitive ability, the multivariate approach provides distinct advantages over univariate intelligence GWAS. First, our analysis aggregates genetic information across 1,940,623 total participants (vs. 110,988 in intelligence GWAS alone), substantially improving statistical power. Second, we identified 275 novel loci (7.2% of total) not reaching genome-wide significance in any constituent univariate GWAS, representing genuinely new genetic discoveries. Third, the latent factor captures cross-trait genetic architecture-genetic variants affecting multiple cognitive domains through shared biological mechanisms-rather than intelligence-specific pathways. Critically, the remaining five phenotypes contribute unique genetic variance (specific factor loadings: Processing Speed 0.398, Educational Attainment 0.718, Executive Function 0.228, Memory Performance 0.551, Reaction Time 0.923), indicating that mvCognitive represents a broader cognitive construct than intelligence alone. The multivariate framework identifies pleiotropic variants whose effects are amplified when analyzed across correlated phenotypes, enabling detection of shared mechanisms missed by single-trait analyses.

### Genomic structural equation model GWAS stratified assessment

By extending structural equation modeling (SEM) to incorporate individual variation, we generated a latent factor GWAS that estimated associations between 1,219,586 single nucleotide polymorphisms (SNPs) and our cognitive ability factor (Supplementary Table 5a). We identified 62 lead SNPs across 1,067 genomic loci at *P* < 5 × 10^–12^, and 33 lead SNPs across 322 genomic loci at the more stringent threshold of *P* < 5 × 10^–16^ (Supplementary Table 5b,c). The newly identified cognitive ability lead SNPs were primarily enriched in pathways related to neurotransmitter metabolism, neurodevelopment, and synaptic plasticity, while also involving metabolic regulation and immune system modulation.

Through systematic literature review, we found that these SNPs included both classical cognitive genes such as rs4820249 (COMT), rs429358 (APOE), and rs6265 (BDNF), which provided important validation in cognitive research, as well as novel discoveries from our cognitive ability structural equation model, including key variant loci such as rs1628294 (ERRFI1), rs17374337 (TSEN2), and rs148729815 (HLA-DQA1) (detailed literature comparison analysis in Supplementary Table 5d). By introducing genomic structural equation modeling (genomic SEM), this study not only validated the role of classical cognitive genes in the comprehensive cognitive model but also discovered genetic variants involving novel pathways such as RNA processing, immune regulation, and signal transduction, providing important insights into the shared genetic foundation of cognitive abilities and potential therapeutic targets.

### Genomic control assessment based on LD score regression

Through the parameter controls described in our methods, we removed a total of 729,630 SNPs and retained 489,956 effective SNPs after regression coefficient filtering. The mean chi-square value across all SNPs was Mean Chi^2^ = 2.234, with genomic control Lambda GC = 1.857, maximum Chi^2^ value = 913.763, and 1,584 genome-wide significant SNPs. Heterogeneity testing passed (intercept approaching 1.0), with total observed-scale heritability (h^2^) = 3.342 × 10^–5^ (1.127 × 10^–6^), ratio of genetic to environmental contributions = 0.0174 (0.0157), regression model intercept = 1.0215, and regression model intercept standard error = 0.0194. Multiple estimates suggested that the potential inflation in our structural equation model was likely due to polygenic heritability signals rather than population stratification bias or pleiotropy parameter effects.

### FUMA-based assessment of cognitive ability structural equation model

**Fig. 3:**
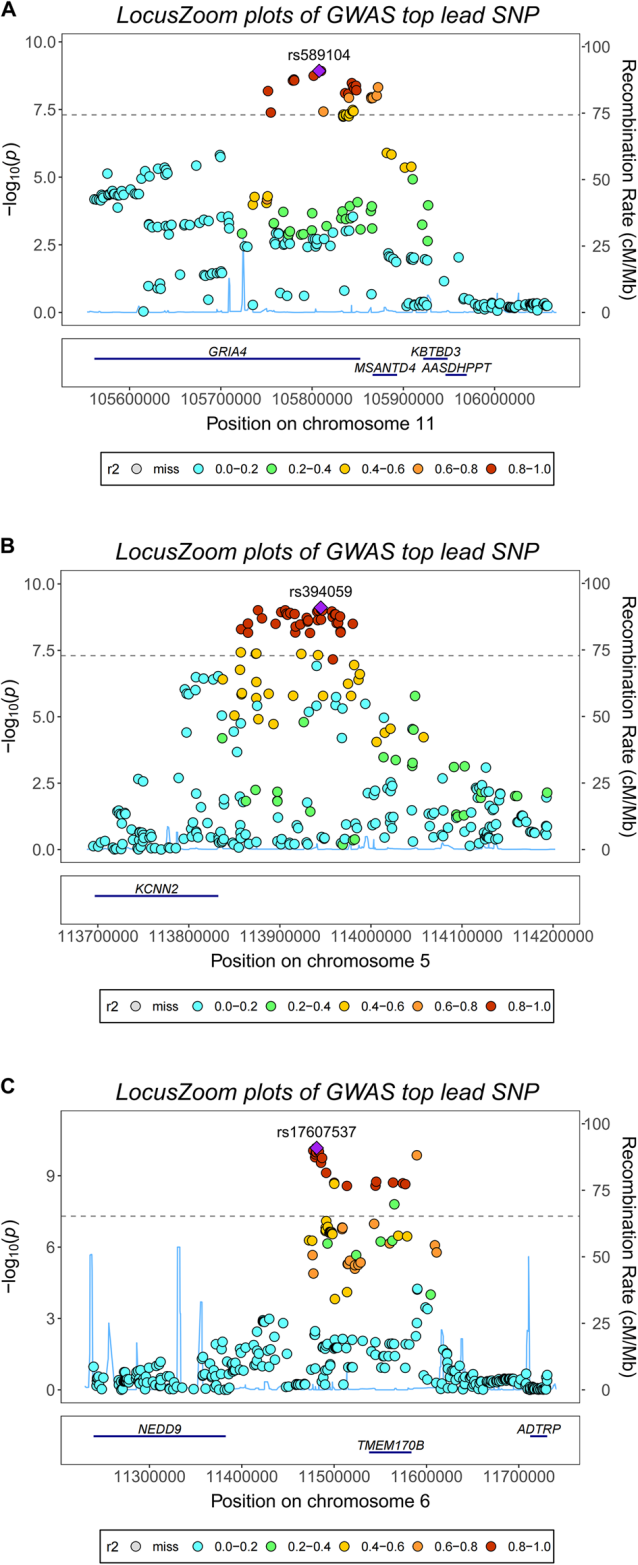

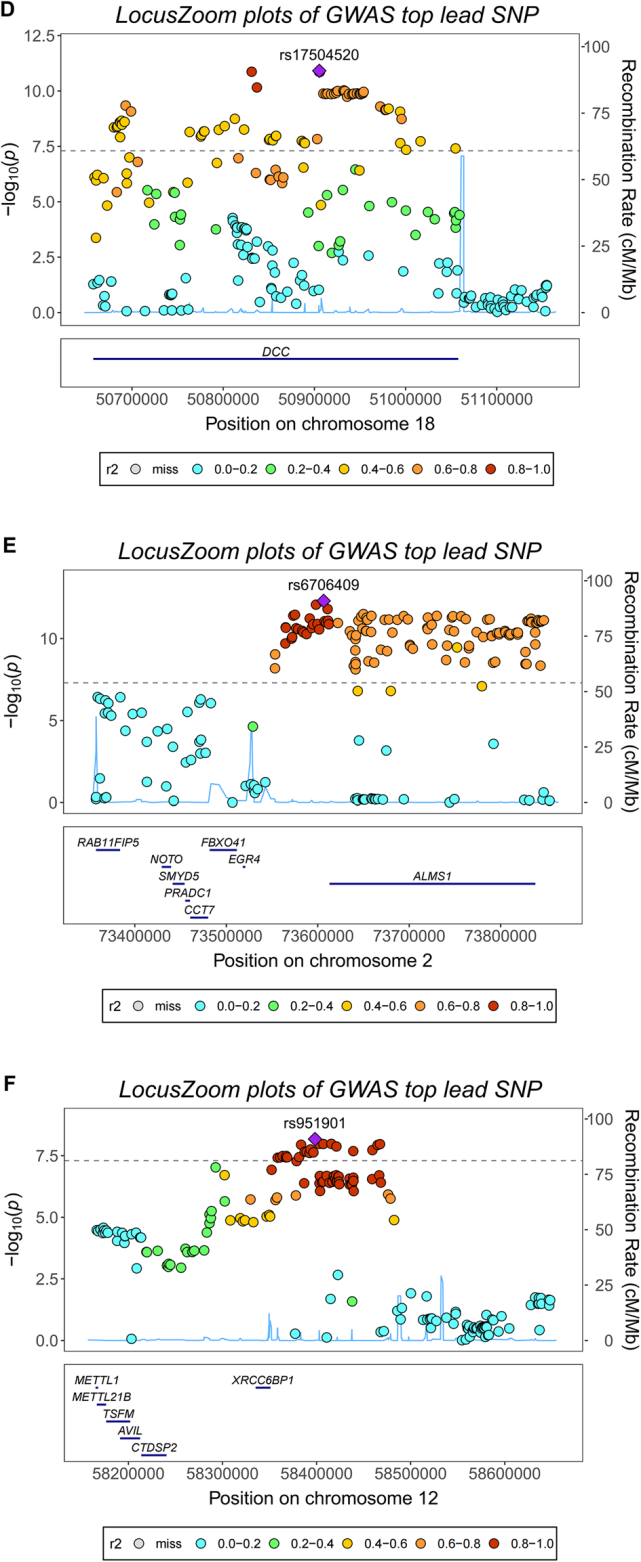
Regional association plots for cognitive ability lead SNPs identified by genomic structural equation modeling. LocusZoom plots displaying regional association signals for six representative lead SNPs from the cognitive ability GWAS. Each panel shows the -log₁₀ (P-value) for SNPs (y-axis) plotted against chromosomal position (x-axis), with recombination rate overlaid as a blue line. Lead SNPs are indicated by purple diamonds, and surrounding SNPs are color-coded according to linkage disequilibrium (LD) with the lead variant (r^2^ values: 0.0–0.2 in cyan, 0.2–0.4 in green, 0.4–0.6 in orange, 0.6–0.8 in orange-red, 0.8–1.0 in red). Gene annotations are shown below each plot. (**A**) rs589104 on chromosome 11 near GRIA4, MSANTD4, and ARS2LIPPT genes. (**B**) rs394059 on chromosome 5 near KCNN2. (**C**) rs17607537 on chromosome 6 near NEDD9, TMEM112B, and ADTRP. (**D**) rs17504520 on chromosome 18 near DCC. (**E**) rs6706409 on chromosome 2 in a gene-rich region including RAB11FIP5, NCD, FBXO41, EGR4, SMG05, PPADC1, CCT7, and ALMS1. (**F**) rs951901 on chromosome 12 near METL1, METL21B, TSEN, AVIL, XRC2C8SP1, and CYP2C cluster. The plots demonstrate clear association peaks with distinct LD patterns supporting the statistical significance of identified lead variants.

**Fig. 4:**
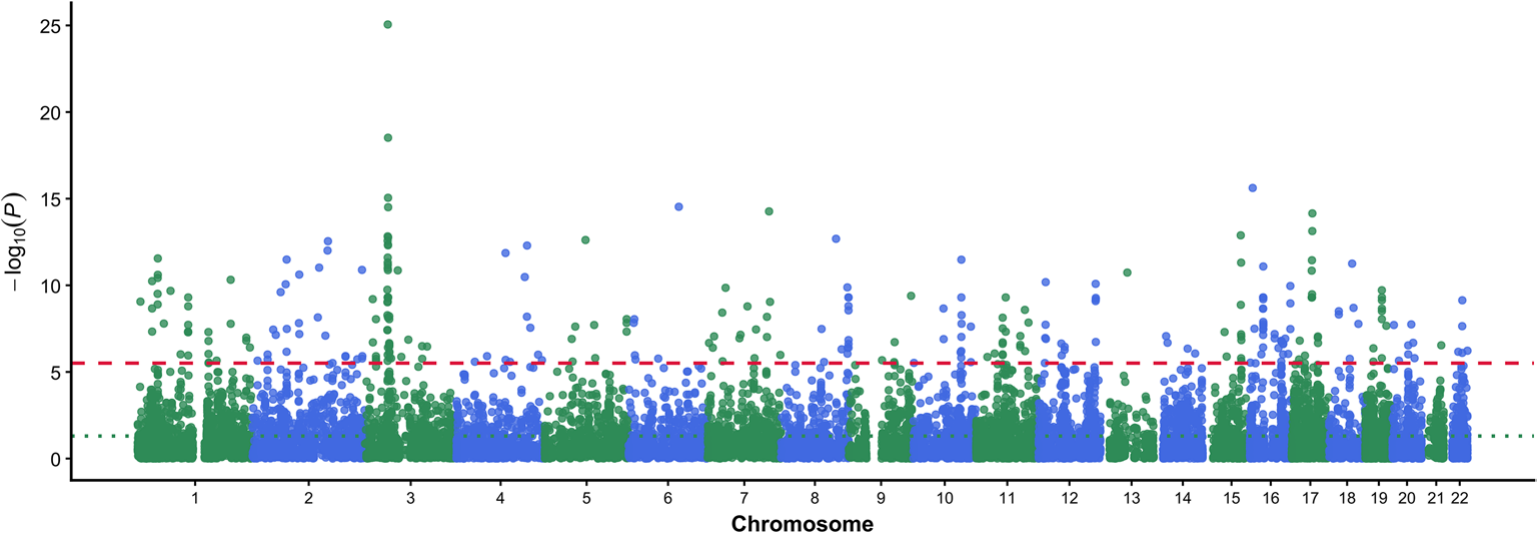
Manhattan plot from FUMA-based assessment of cognitive ability structural equation model. Manhattan plot displaying MAGMA gene-based association results for cognitive abilities. The analysis tested 16,129 protein-coding genes using MAGMA implemented in FUMA software. The x-axis shows chromosomal positions (chromosomes 1–22), and the y-axis represents -log₁₀ (*P*-values) for gene-level association strength. Points are color-coded alternately (green and blue) by chromosome. The red dashed line indicates the Bonferroni-corrected significance threshold (*P* = 0.05/16,129 = 3.1 × 10^–6^, corresponding to -log₁₀ (*P*) ≈ 5.5), accounting for multiple testing across all genes analyzed. The green dotted line shows nominal significance (*P* = 0.05, corresponding to -log₁₀ (*P*) ≈ 1.3). The analysis identified 303 genes passing the Bonferroni-corrected threshold, which were used for subsequent pathway enrichment analysis. Gene-level associations complement the 206 independent genomic risk loci identified in SNP-level analysis.

Using FUMA software for genomic structural equation assessment, we identified 206 risk gene loci (Table 5E, Fig. 3) and detected 2,014 potential cognitive ability-related genes under genome-wide significant control (sig.thres = 5 × 10^–8^, FDR < 0.05, Fig. 4). Through FUMA, we annotated 225 lead SNP loci, with the majority located in intergenic regions (Supplementary Table 6).

GWAS subtraction analysis identified 131 loci previously reported in educational attainment GWAS (such as rs12735232, rs11210887, etc.) (Supplementary Table 7). Comparison with previous literature revealed important discovery patterns for these loci: for example, rs12735232 was reported in educational attainment and reaction time-related research literature, but we found that this locus is not directly causative for educational attainment phenotype, but rather a potential mediating locus that indirectly affects educational achievement by influencing basic cognitive abilities (such as processing speed, executive function, memory performance, and intelligence). Rs11210887 showed consistency with our studied cognitive ability phenotypes in most previous educational attainment literature, further confirming the hypothesis that cognitive ability serves as the biological foundation for educational achievement (Supplementary Tables 8, 9).

### Fine-mapping analysis

**Fig. 5:**
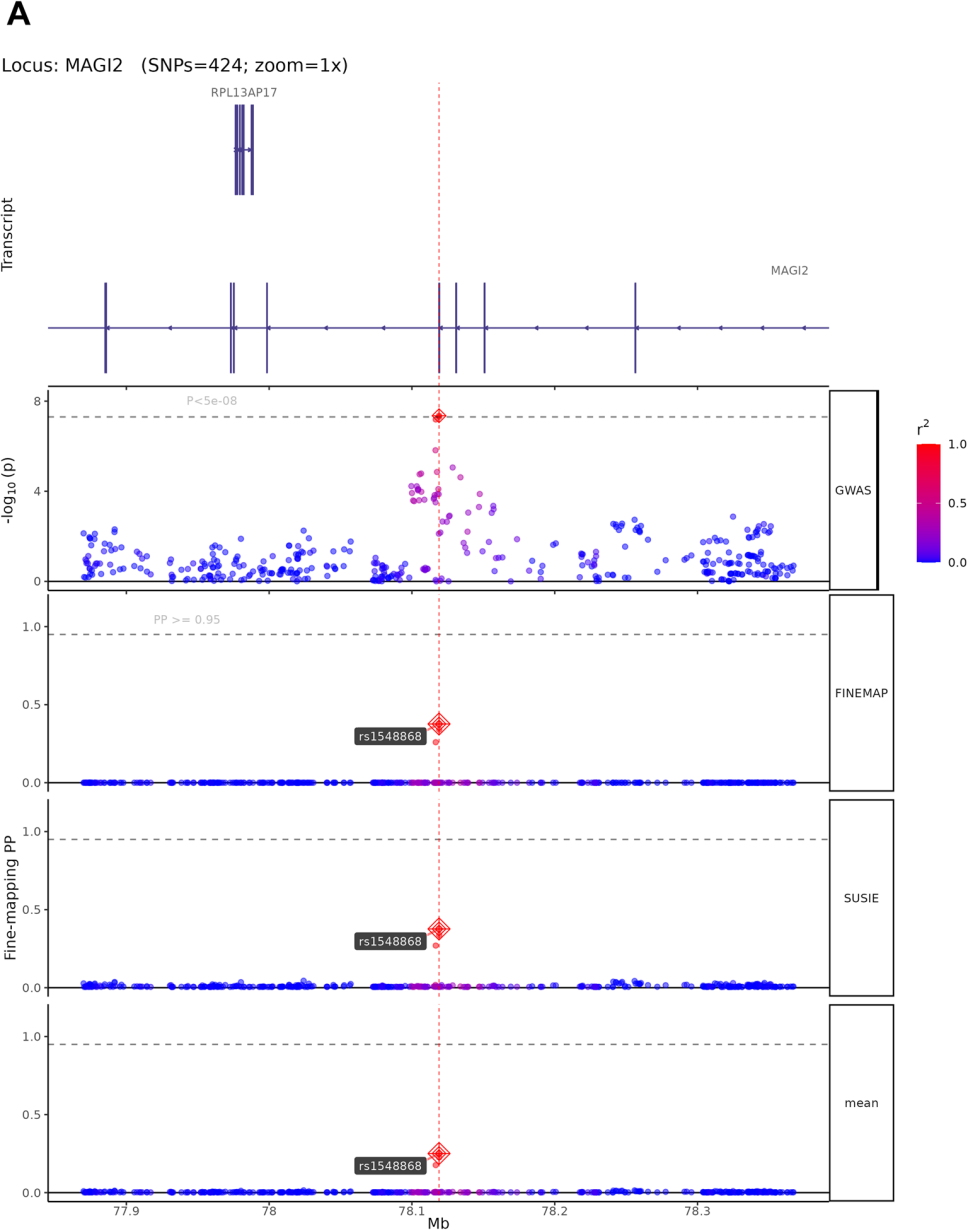

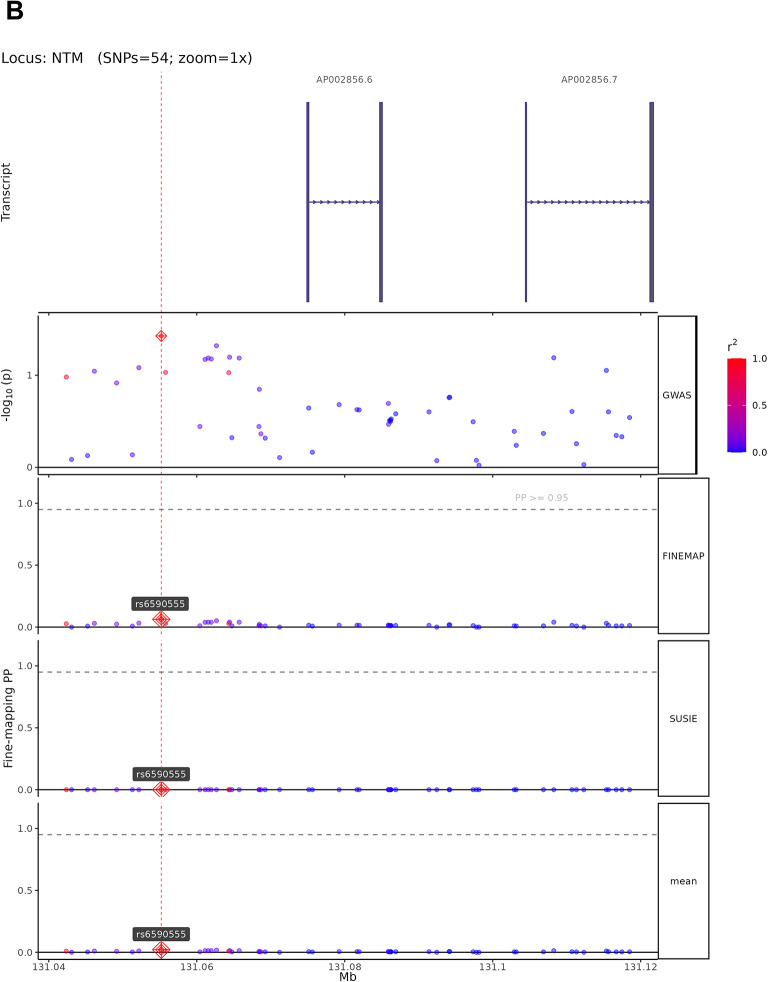

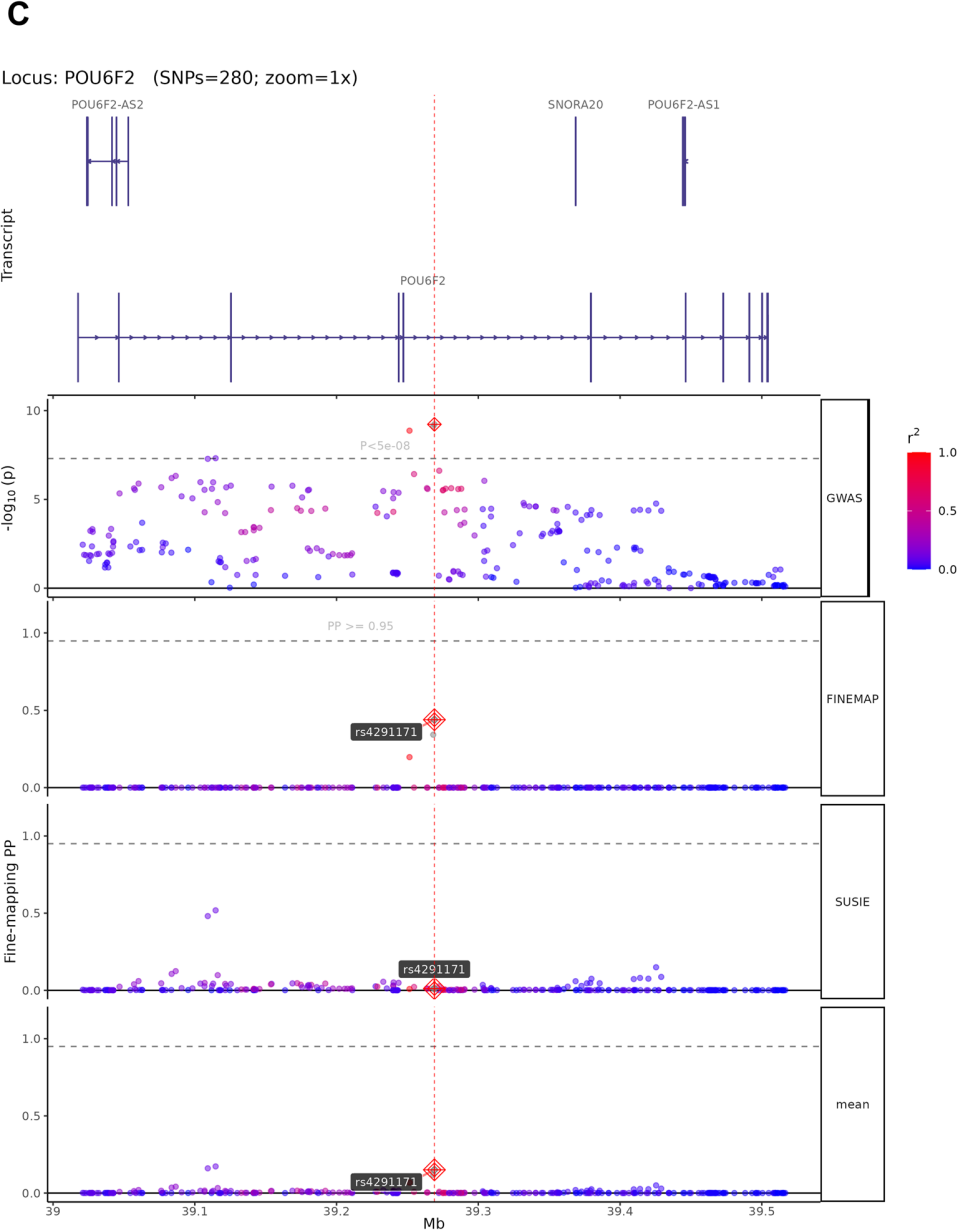

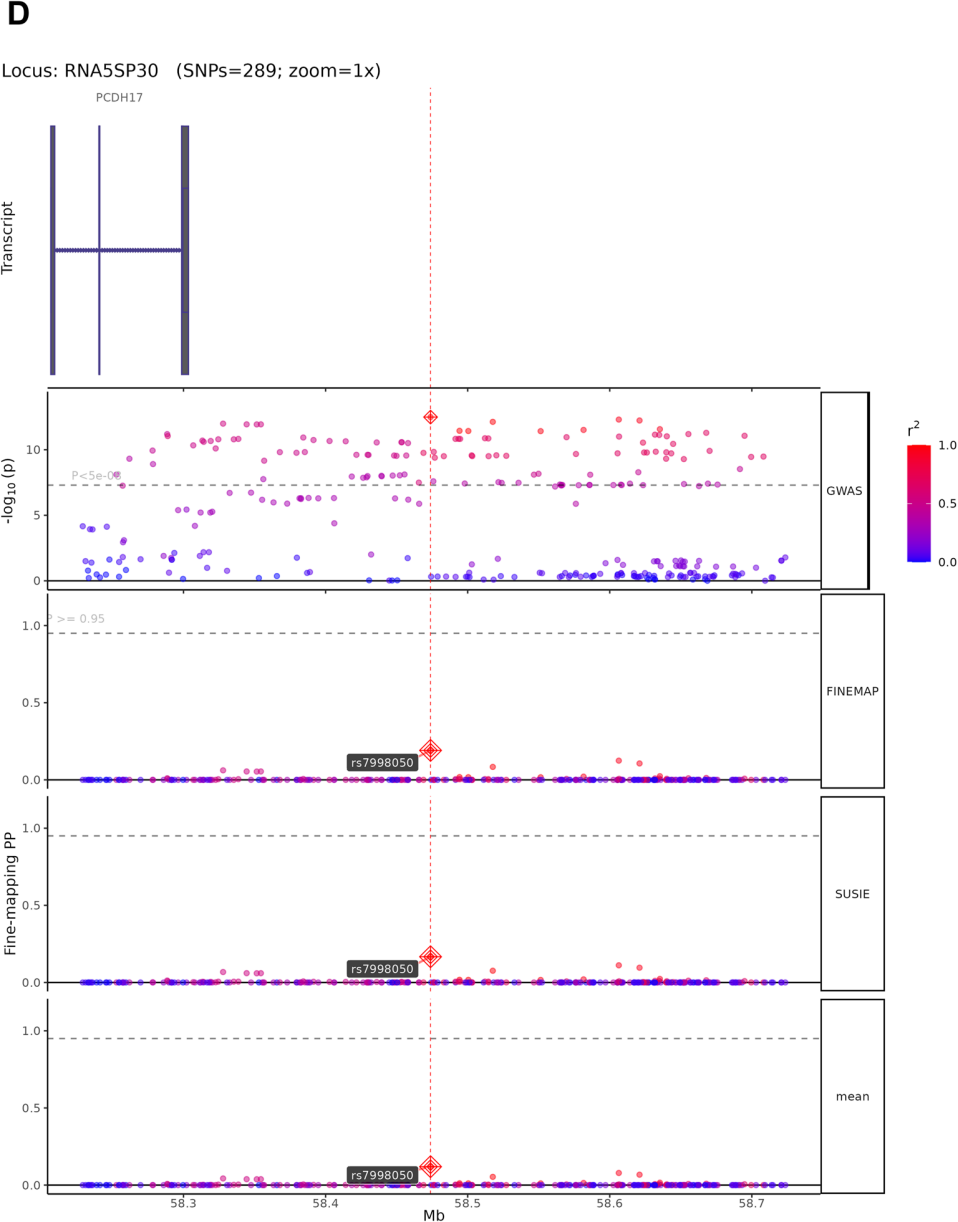
Fine-mapping analysis of high-confidence candidate causal variants from cognitive ability GWAS. (**A**–**D**) Fine-mapping results using SuSiE and FINEMAP methods for four representative genomic loci. Each panel displays (from top to bottom): gene transcript structures, regional GWAS association plot, FINEMAP posterior probabilities (PP), SuSiE posterior probabilities, and mean PP across both methods. SNPs are color-coded by linkage disequilibrium (LD) with the lead variant (r^2^scale: 0.0 in blue to 1.0 in red). Lead SNPs with PP > 0.95 are marked with red diamonds and labeled. Horizontal dashed lines indicate the PP = 0.95 threshold for high-confidence candidate causal variants. (**A**) MAGI2 locus (424 SNPs analyzed): Fine-mapping identifies rs1548868 as a candidate causal variant (mean PP > 0.95) near RPL13AP17 transcript, with convergent evidence from both FINEMAP and SuSiE methods. (**B**) NTM locus (54 SNPs analyzed): rs6590555 identified as the most probable causal variant in this credible set, located near NTM gene transcripts AP002856.6 and AP002856.7. (**C**) POU6F2 locus (280 SNPs analyzed): rs4291171 prioritized as a high-confidence candidate causal variant (PP > 0.95) within the POU6F2 gene region, flanked by POU6F2-AS2, SNORA20, and POU6F2-AS1 transcripts. (**D**) RNA5SP30 locus (289 SNPs analyzed): rs7998050 shows high posterior probability (mean PP > 0.95) near PCDH17 transcript, with consistent support from both fine-mapping algorithms. These fine-mapping results narrow genome-wide significant associations to small credible sets of likely causal variants, facilitating downstream functional validation.

The 13 high-confidence candidate causal variants map to genes with established roles in neural development and synaptic function. The four representative loci shown in Fig. 5 exemplify diverse biological mechanisms: rs1548868 (MAGI2 locus) maps near MAGI2 (membrane-associated guanylate kinase inverted 2), a scaffolding protein enriched at excitatory synapses that organizes postsynaptic signaling complexes and regulates synaptic plasticity. rs6590555 (NTM locus) localizes to neurotrimin, a GPI-anchored neuronal cell adhesion molecule critical for neurite outgrowth and synapse formation during brain development. rs4291171 (POU6F2 locus) lies within POU class 6 homeobox 2, a transcription factor essential for neuronal subtype specification and forebrain development. rs7998050 (RNA5SP30/PCDH17 locus) is near protocadherin 17, a member of the cadherin superfamily that establishes neural circuit specificity through cell–cell recognition. Among the remaining nine variants, rs4820249 on chromosome 22 maps to COMT (catechol-O-methyltransferase), regulating prefrontal dopamine metabolism—a well-established cognitive mechanism validating our approach. The convergence on synaptic scaffolding (MAGI2), cell adhesion (NTM, PCDH17), transcriptional regulation (POU6F2), and neurotransmitter metabolism (COMT) indicates that cognitive genetic architecture operates through multiple coordinated biological pathways. Complete functional annotations for all 13 variants are provided in Supplementary Table 10.

### Transcriptomic prediction

**Fig. 6:**
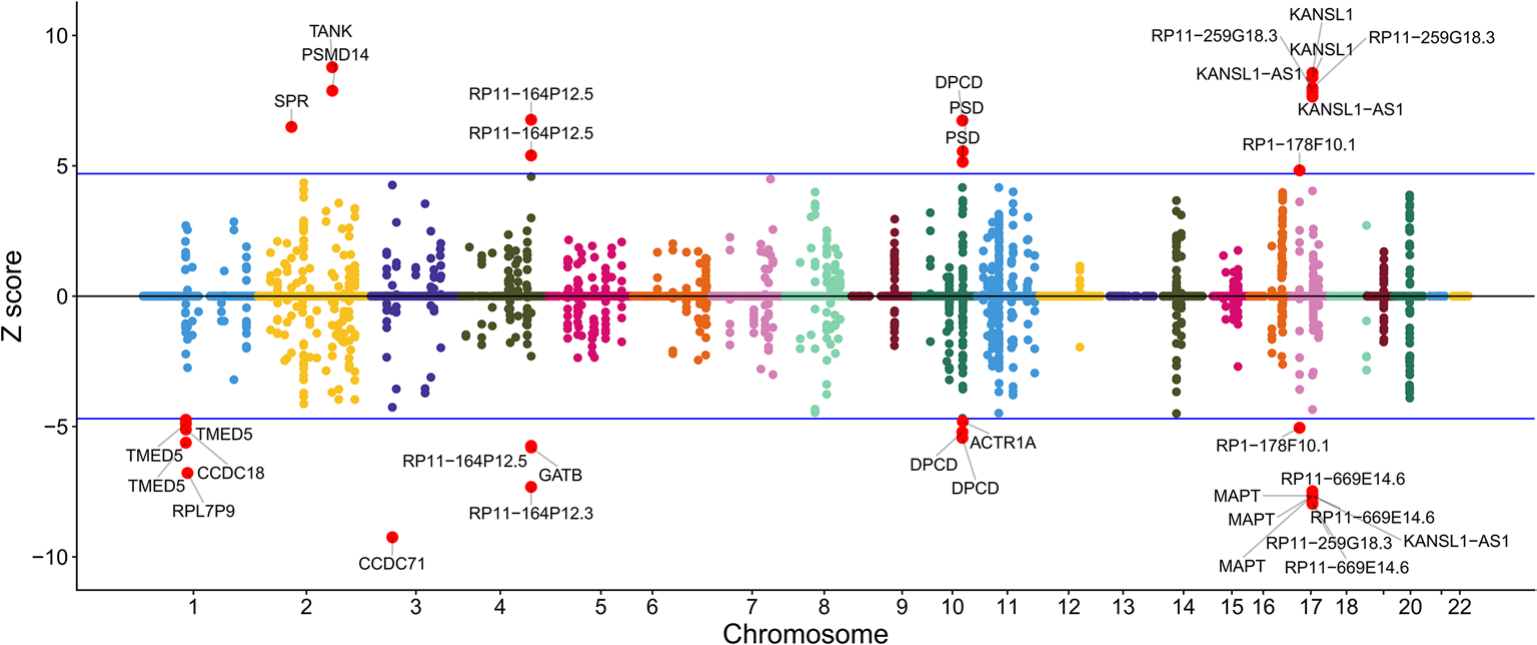
Transcriptome-wide association study (TWAS) results for cognitive abilities. Manhattan plot showing TWAS Z-scores across chromosomes 1–22. Each point represents a gene-tissue pair from GTEx v.8; the same gene may appear multiple times with tissue-specific associations. Positive Z-scores indicate genes whose upregulation associates with increased cognitive abilities; negative Z-scores indicate genes whose downregulation associates with increased cognitive abilities. Blue horizontal lines mark significance threshold (|Z|> 4.9, P < 0.05 after correction). Red circles highlight significant genes. Significant positive associations: TANK, PSMD14, SPR (chr2); RP11-164P12.5 (chr4); DPCD, PSD (chr10-11); KANSL1, KANSL1-AS1, RP11-259G18.3, RP1-178F10.1 (chr17).Significant negative associations: TMED5, CCDC18, RPL7P9 (chr1); CCDC71, RP11-164P12.3 (chr3); GATB, RP11-164P12.5 (chr4); ACTR1A, DPCD (chr10); MAPT, KANSL1-AS1, RP11-669E14.6 (chr17).Analysis identified 33 genes exceeding significance threshold. TWAS used FUSION with GTEx v.8 weights (37,920 gene/tissue pairs).

Next, we conducted transcriptome-wide association study (TWAS) using FUSION to identify gene-level associations related to cognitive ability genetic features. Applying stringent Bonferroni correction (*P* < 1.32 × 10^–6^ for 37,920 gene-tissue pairs tested), we identified 33 high-confidence genes that exceeded the transcriptome-wide significance threshold (Extended Data Table 1, Fig. 6). These represent priority candidates for functional follow-up. Additionally, 13,394 gene-tissue pairs showed nominal significance (*P* < 0.05), which we retained for pathway enrichment analyses and cross-method validation (see below). Subsequently, FOCUS fine-mapping analysis of the genomic structural equation data revealed 179 genes that showed potential causal signals with cognitive abilities. To identify genes with convergent evidence across methods, we performed intersection analysis between TWAS and FOCUS results. Among the high-confidence candidates, genes such as TANK and KANSL1 had TWAS Z-scores > 0, indicating that predicted gene expression was positively correlated with cognitive abilities, suggesting that upregulation of these genes may be associated with increased cognitive performance. Conversely, genes including CCDC152, ITGAV, SLC22A3, and MRPL33 had TWAS Z-scores < 0, indicating that their downregulation was associated with increased cognitive abilities (TWAS and FOCUS intersection results in Supplementary Table 11).

### Pathway, cell type, and Mendelian disease gene enrichment

For functional enrichment analyses, we used the 303 genes identified by MAGMA at genome-wide significant threshold (Bonferroni-corrected *P* < 3.1 × 10^–6^) as the primary gene set, as these represent genome-wide significant associations with well-controlled family-wise error rate.

Multi-marker Analysis of GenoMic Annotation (MAGMA) genomic mapping identified 303 genes (Supplementary Table 12), which we used for gene set analysis. These genes showed significant enrichment in GSEA entries (Supplementary Table 13). The most significant enrichment signals were concentrated in neurodevelopmental pathways, including deletion syndrome-related gene sets (*P* = 1.53 × 10^–8^), synapse organization and structure-related gene sets (such as GOBP_SYNAPSE_ORGANIZATION, *P* = 1.41 × 10^–10^), and neuronal development and morphogenesis-related pathways. Additionally, these genes were significantly enriched in multiple cognitive ability-related GWAS signals, including intelligence (*P* = 1.49 × 10^–102^), general cognitive ability (*P* = 9.82 × 10^–86^), and educational attainment (*P* = 1.20 × 10^–31^), further validating the association between our identified genetic variants and cognitive function.

**Fig. 7:**
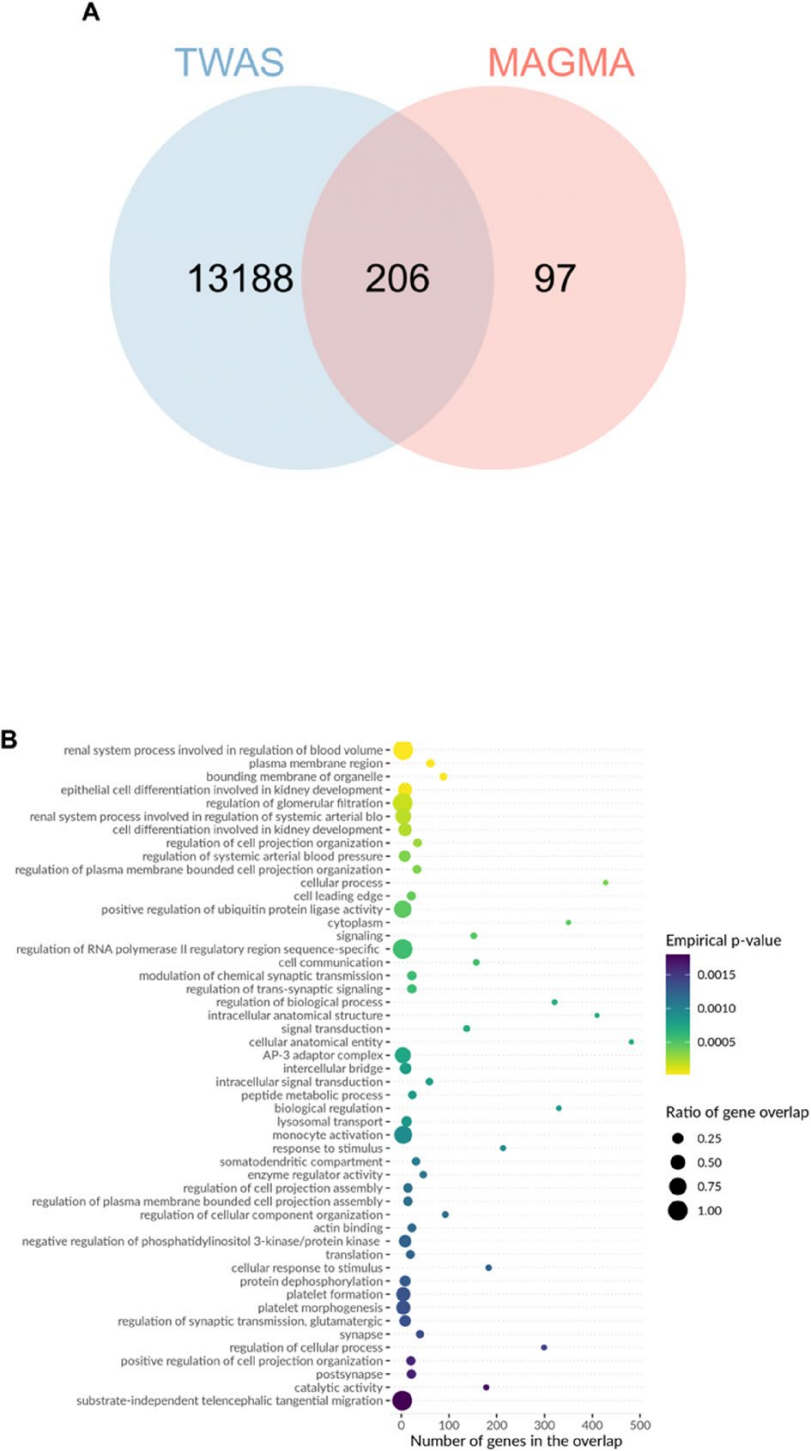

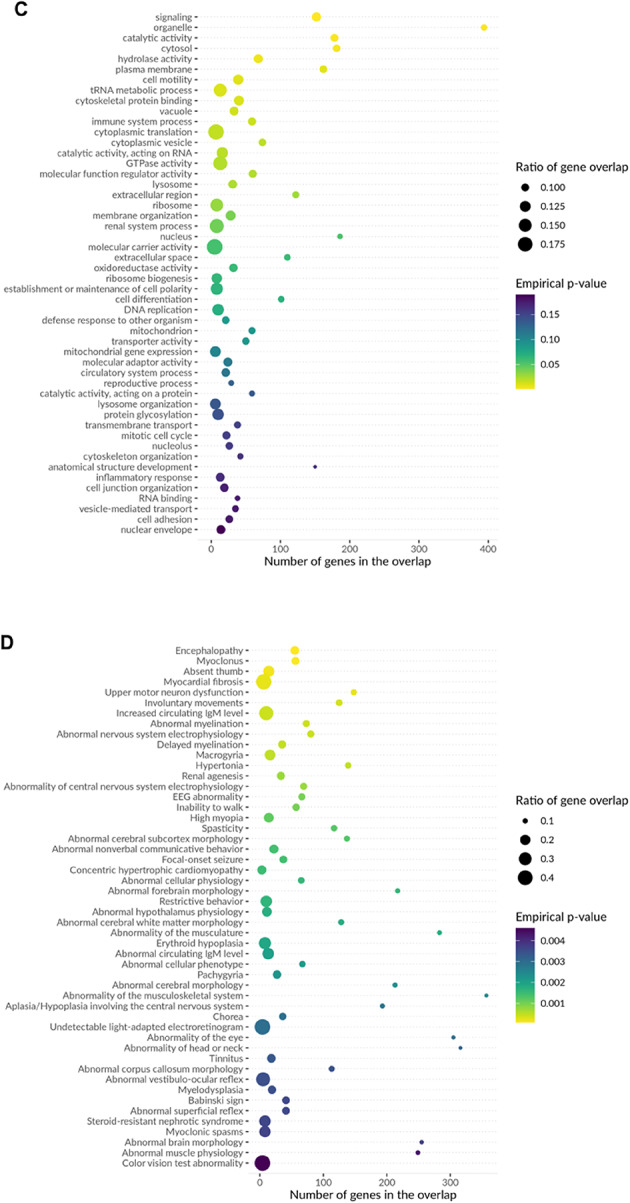

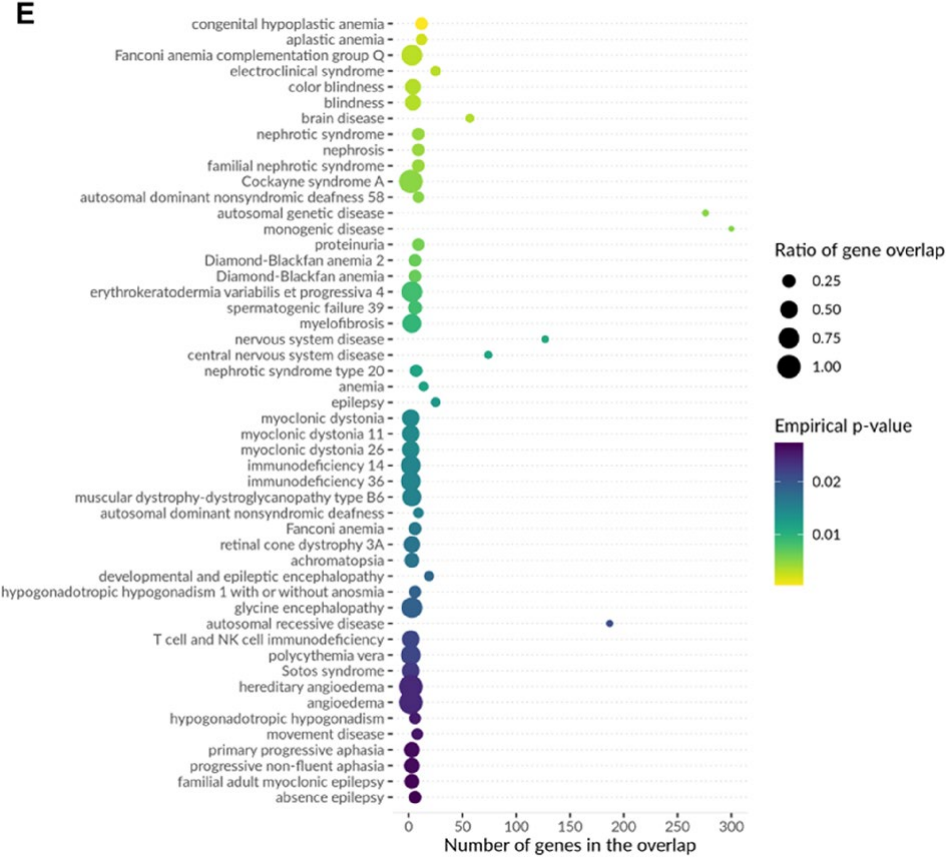
TWAS-MAGMA intersection analysis and functional enrichment of cognitive ability-associated genes. (**A**) Venn diagram showing overlap between TWAS analysis (13,394 gene-tissue pairs with nominal P < 0.05 from 37,920 tested) and MAGMA analysis (303 genes at Bonferroni-corrected *P* < 3.1 × 10⁻⁶ from 16,129 tested). The 206 overlapping genes (68.0% of MAGMA genes) demonstrate convergence between transcriptomic and genomic approaches. TWAS identified 33 genes at stringent threshold (*P* < 1.32 × 10⁻⁶); these are a subset of the 13,394 shown here. The 206 genes validated transcriptomic support for MAGMA findings; the 303 MAGMA genes were used for enrichment analyses (panels B-E). (**B**) Biological process enrichment showing significant associations with neural system processes, synaptic transmission, cell differentiation, protein localization, and metabolic processes. Top enrichments include regulation of synaptic transmission (> 300 genes), cell differentiation and developmental processes (~ 200–250 genes), and various metabolic and transport processes. (**C**) Additional biological processes highlighting cytoskeletal organization, membrane transport, catalytic activity regulation, and cellular organization processes. (**D**) Disease ontology enrichment revealing associations with encephalopathy, myoclonus, ataxia, muscular disorders, nervous system abnormalities, and various neurological conditions. Notable enrichments include upper motor neuron dysfunction, abnormal nervous system electrophysiology, and neurodevelopmental disorders.Color intensity represents FDR-corrected *P*-values (Benjamini–Hochberg method applied to empirical *P*-values from permutation testing). All displayed associations meet FDR < 0.05 significance threshold. (**E**) Molecular function and additional pathway enrichments showing associations with enzymatic activities, binding functions, transport activities, and regulatory processes. The analysis demonstrates that cognitive ability-associated genes are significantly enriched in neurodevelopmental pathways, synaptic function, and neurological disease-related processes, supporting their functional relevance to cognitive phenotypes.

To validate transcriptomic evidence for MAGMA-identified genes, we compared the 303 genes from MAGMA (Bonferroni-corrected P < 3.1 × 10^–6^) with the 13,394 gene-tissue pairs from TWAS (nominal *P* < 0.05). Results showed 206 genes were detected in both analyses (Fig. 7A), indicating significant associations at both genomic (MAGMA) and transcriptomic (TWAS) levels. This high overlap validates the effectiveness of multivariate GWAS in identifying functionally relevant genes. We used nominal significance threshold for TWAS (n = 13,394) rather than stringent threshold (n = 33) to enable comprehensive cross-method validation (Fig. 7).

Furthermore, biological processes mapped by MendelVar enrichment were also supported by GSEA entries. Enrichment analysis revealed significant signals at multiple levels: disease enrichment analysis showed that cognitive-related genes were significantly enriched in blood system diseases and nervous system diseases (FDR < 0.005); biological process enrichment primarily involved renal system blood volume regulation and cell differentiation processes (FDR < 0.0005); molecular function enrichment focused on signal transduction, organelle function, and other basic molecular processes (FDR < 0.05); phenotype enrichment analysis confirmed associations with encephalopathy, myoclonus, and other neurodevelopmental abnormalities (FDR < 0.001). These enrichment results strongly support the important role of cognitive ability-related genes in neurodevelopment, blood systems, and basic cellular functions.

In cell type enrichment analysis from different datasets, we conducted systematic validation using multiple datasets. In the primary Tabula Muris analysis, 5 cell types exceeded the significance threshold after multiple comparison correction (FDR < 0.05) (Supplementary Table 14). The top two cell types were Brain_Non-Myeloid_neuron (*P* = 3.55 × 10^–12^) and Brain_Non-Myeloid_oligodendrocyte_precursor_cell (*P* = 3.73 × 10^–6^).

**Fig. 8:**
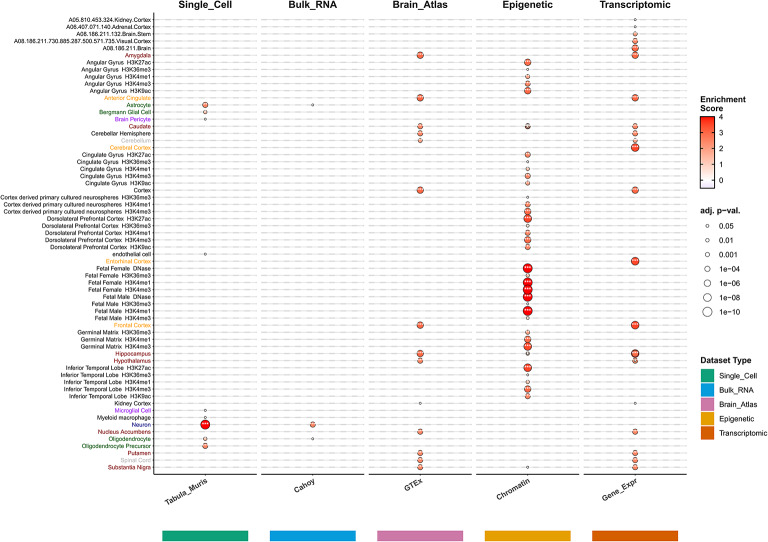
Cell-type and tissue-specific enrichment analysis of cognitive ability-associated genes. Dot plot showing enrichment analysis across five datasets: Tabula Muris (single-cell), Cahoy (bulk RNA), GTEx (brain atlas), Chromatin (epigenetic), and Gene Expression (transcriptomic). Point size indicates enrichment score magnitude, and color intensity represents statistical significance (adjusted P-values from white P > 0.05 to dark red P < 1 × 10⁻^1^⁰). Key findings: Neuron shows strongest enrichment in single-cell data (P = 3.55 × 10⁻^12^), confirming central role of neuronal function. Oligodendrocyte Precursor cells demonstrate significant enrichment (P = 3.73 × 10⁻⁶), supporting white matter plasticity contributions. Multiple brain regions show significant enrichment including Angular Gyrus, Cingulate Gyrus, Dorsolateral Prefrontal Cortex, Inferior Temporal Lobe, and Germinal Matrix. Fetal brain tissues exhibit strong enrichment in epigenetic datasets, suggesting critical neurodevelopmental windows. GTEx analysis reveals enrichment across 13 brain tissues (FDR < 0.05). Results demonstrate cognitive ability genes are predominantly expressed in neural tissues with cell-type specificity consistent with neurodevelopmental and synaptic functions.

**Fig. 9:**
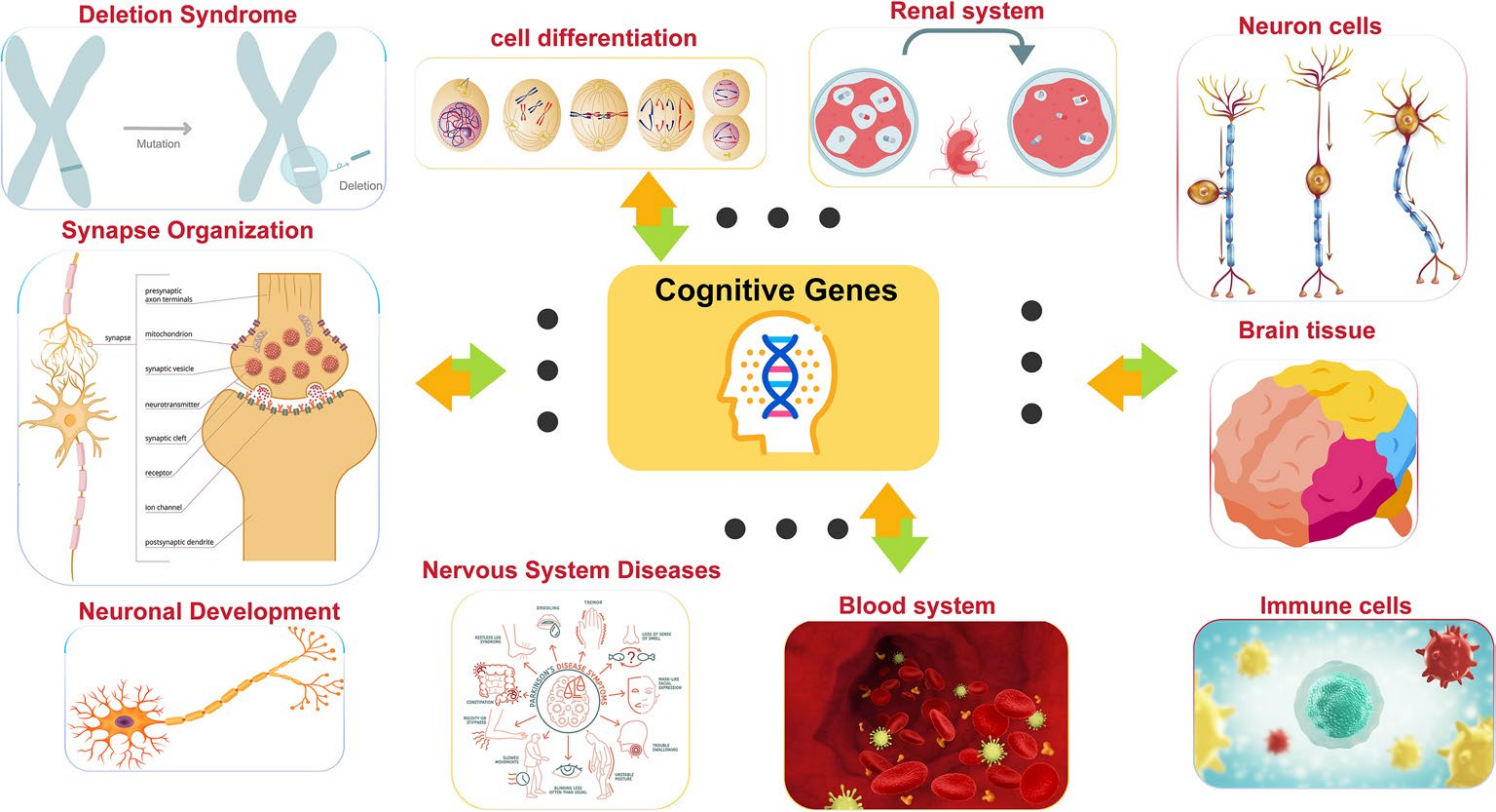
Systems-level associations of cognitive ability genes across biological domains. Conceptual diagram illustrating the multi-system biological connections of cognitive ability-associated genes identified through genomic structural equation modeling. The central Cognitive Genes hub (yellow box with DNA helix) represents the 206 high-confidence genes identified through TWAS-MAGMA intersection analysis. Orange and green arrows indicate directional associations between cognitive genes and distinct biological systems and disease categories. Connected biological systems include: Deletion Syndrome (upper left) showing chromosomal deletions and mutations relevant to neurodevelopmental disorders; Cell Differentiation (upper center) illustrating cellular development processes from progenitor to mature cell types; Renal System (upper right) representing kidney function and metabolic regulation; Neuron Cells (right) displaying various neuronal morphologies including pyramidal, bipolar, and multipolar neurons; Brain Tissue (lower right) showing anatomical brain regions with distinct functional areas; Immune Cells (lower right) representing various immune cell types and inflammatory responses; Blood System (lower center) illustrating cardiovascular components and hematological processes; Nervous System Diseases (lower center) showing disease network connections and pathological states; Neuronal Development (lower left) depicting developmental stages of neuronal maturation; and Synapse Organization (left) detailing synaptic structure components including presynaptic terminals, synaptic vesicles, neurotransmitters, receptors, and postsynaptic dendrites. This systems-level view demonstrates that cognitive ability genes operate through interconnected biological networks spanning neurodevelopment, synaptic function, immune regulation, metabolic processes, and disease susceptibility, supporting the concept that cognition emerges from coordinated multi-system interactions rather than isolated neural processes.

Further validation analyses showed: In Cahoy brain cell data, Neuron cell type was significantly enriched (*P* = 8.31 × 10^–5^, FDR = 2.49 × 10^–4^); In GTEx tissue analysis, 13 brain tissues were all significantly enriched (FDR < 0.05), with Brain_Frontal_Cortex showing the strongest enrichment (*P* = 1.84 × 10^–8^); In multi-tissue gene expression analysis, 18 brain-related tissues/cell types were significantly enriched, further confirming the strong association between cognitive abilities and the nervous system. Cognitive abilities also showed enrichment trends in B cells/immune cell precursors, with 6 immune-related cell types having *P* < 0.05, including Marrow_naive_B_cell, Marrow_macrophage, Spleen_B_cell, etc., although they did not reach significance thresholds after multiple comparison correction. These consistent cross-dataset results strongly support the conclusion that cognitive abilities primarily function through brain cell-specific mechanisms (Figs. 8, 9).

### Heritability contribution results from genomic regions

We found that cognitive ability-related genetic contribution loci were significantly concentrated in regulatory regions and functionally enriched areas of chromosomes. LDSC partitioned heritability analysis based on 53 functional genomic regions showed that these regions primarily involved key sites for gene expression regulation, chromatin modification, and transcription factor binding. The effects of genetic variants were most significant in evolutionarily conserved sequences and transcriptional regulatory regions.

Evolutionarily conserved sequence core regions exhibited extremely strong 17.79-fold enrichment (*P* = 9.61 × 10^–18^), while transcription start site core regions achieved 5.97-fold enrichment (*P* = 2.51 × 10^–4^). Active transcription marks H3K9ac and H3K4me3 peak regions showed 5.24-fold and 4.79-fold enrichment, respectively, and enhancer mark H3K4me1 peak regions reached 2.59-fold enrichment. These findings indicate that evolutionarily conserved transcriptional regulatory sites are core carriers of cognitive ability genetic variants and may exert important effects on cognitive phenotypes through regulation of gene expression levels.

**Fig. 10:**
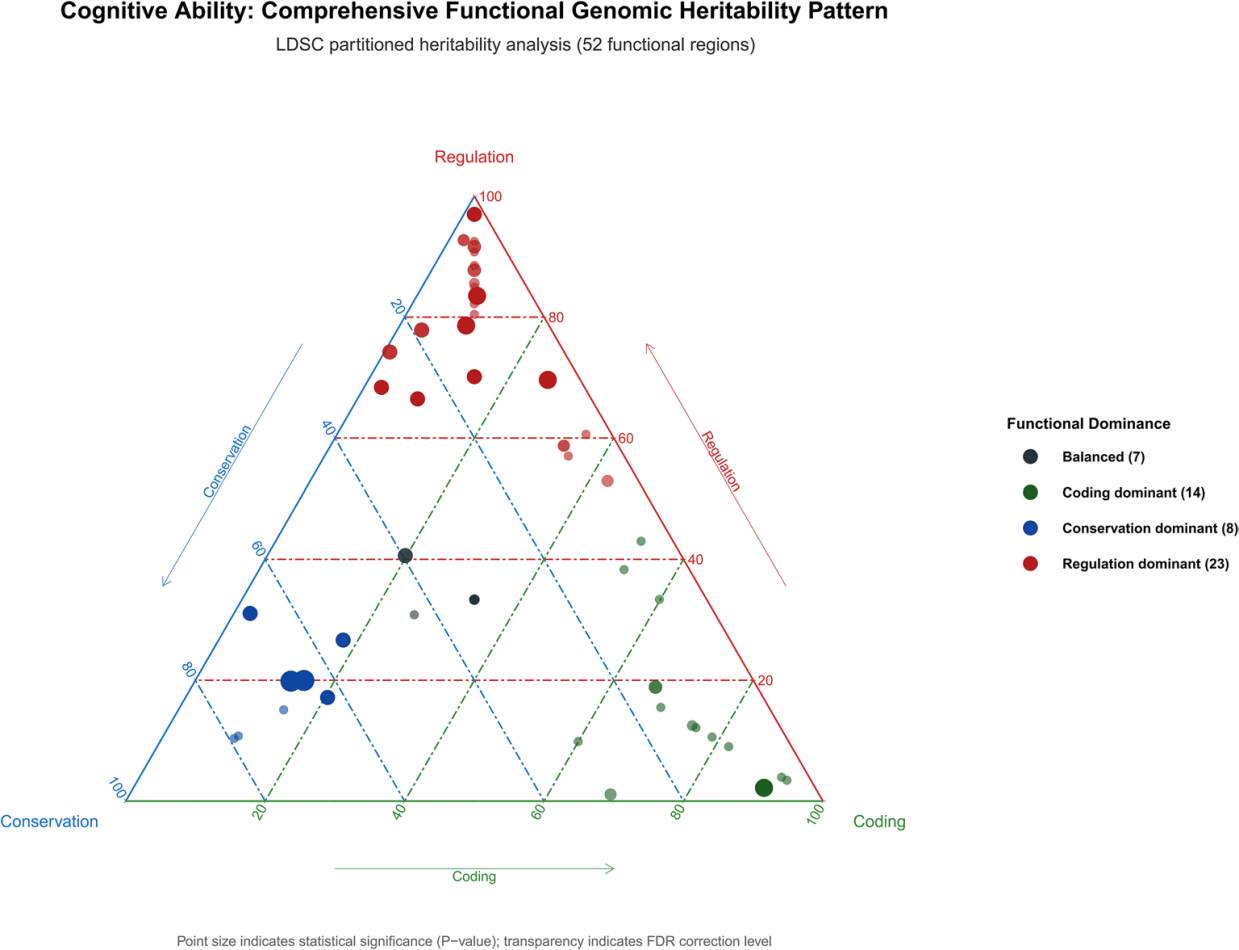
Comprehensive functional genomic heritability pattern of cognitive abilities. Ternary plot displaying LDSC partitioned heritability analysis across 52 functional genomic regions, illustrating the distribution of cognitive ability-associated genetic variants across three major functional categories: Regulation (transcriptional and epigenetic regulatory elements), Conservation (evolutionarily conserved sequences), and Coding (protein-coding regions). Each point represents a specific functional annotation, with position determined by the relative contribution of each functional category. Point size indicates statistical significance (P-value), and transparency indicates FDR correction level. Functional dominance categories are color-coded: Regulation dominant (red, n = 23) representing annotations primarily enriched in regulatory elements; Coding dominant (green, n = 14) representing annotations enriched in protein-coding sequences; Conservation dominant (blue, n = 8) representing annotations enriched in evolutionarily conserved regions; and Balanced (black, n = 7) representing annotations with relatively equal contributions across categories. Key findings: The majority of cognitive ability-associated variants cluster toward the Regulation apex, with 23 functional annotations showing regulatory dominance, indicating that cognitive abilities are primarily influenced by genetic variants affecting gene expression regulation rather than protein-coding changes. Evolutionarily conserved sequence core regions show extreme enrichment (17.79-fold, P = 9.61 × 10⁻^18^), positioned near the Conservation apex. Several annotations show balanced contributions across functional categories, suggesting complex regulatory mechanisms. The distribution pattern demonstrates that cognitive genetic architecture is predominantly driven by regulatory variants in evolutionarily important genomic regions, supporting the “regulatory evolution” model of cognitive trait development.

Additionally, coding regions and certain non-coding regions also showed important genetic contributions. Protein-coding regions displayed 11.29-fold enrichment, 5’ UTR regions achieved 8.00-fold enrichment, while extended intronic regions showed 1.32-fold enrichment at extremely significant levels (*P* = 2.92 × 10^–8^), suggesting that these regions may participate in complex genetic mechanisms through regulation of gene expression or function. This differential contribution pattern between coding and regulatory regions further confirms that the genetic foundation of cognitive abilities relies more on fine control of gene expression regulation (Supplementary Table 15, Fig. 10).

### Multilevel biological foundations of cognitive abilities

**Fig. 11:**
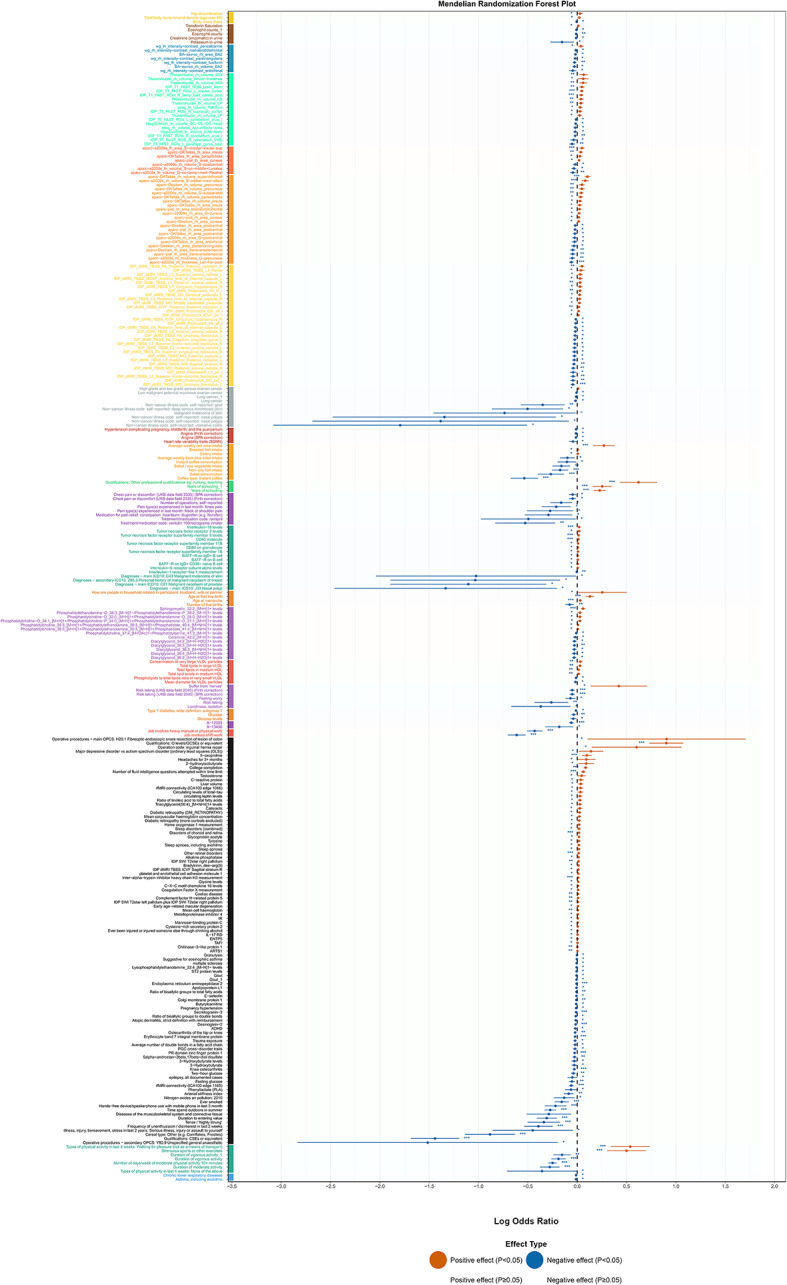
Phenome-wide association analysis of cognitive abilities. Forest plot showing odds ratios (OR) and 95% confidence intervals for cognitive ability associations with human phenotypes. 857 significant phenotypes identified from 50,033 total phenotypes (FDR < 0.05), with 156 protective and 131 risk associations. Vertical dashed line indicates OR = 1.0 (no effect). Key positive associations: Years of education (OR = 1.256, P = 4.63 × 10⁻^14^), university degree completion (OR = 1.089, P = 3.55 × 10⁻^17^). Protective effects: ADHD (OR = 0.980, P = 0.005), moderate intensity activity (OR = 0.757, P = 3.26 × 10⁻^8^), vigorous activity (OR = 0.827, P = 4.32 × 10⁻^7^), fasting glucose (OR = 0.948, P = 6.95 × 10⁻^4^), postprandial glucose (OR = 0.962, P = 1.12 × 10⁻^3^).

We identified 857 phenotypes significantly associated with cognitive abilities from the 50,033 phenotypes in the IEU database (FDR < 0.05). Effect direction distribution revealed 156 protective associations and 131 risk associations. Among these, years of education showed a strong positive association (OR = 1.256, *P* = 4.63 × 10^–14^), and university degree completion was significantly associated (OR = 1.089, *P* = 3.55 × 10^–17^). Cognitive abilities showed protective effects against ADHD (OR = 0.980, *P* = 0.005). Physical activity phenotypes exhibited strong protective associations, including moderate intensity activity duration (OR = 0.757, *P* = 3.26 × 10^–8^) and vigorous activity duration (OR = 0.827, *P* = 4.32 × 10^–7^). Glucose metabolism-related indicators showed beneficial effects, with fasting glucose (OR = 0.948, *P* = 6.95 × 10^–4^) and 2-h postprandial glucose (OR = 0.962, *P* = 1.12 × 10^–3^) both showing protective associations (Supplementary Table 16, Fig. 11).

**Fig. 12:**
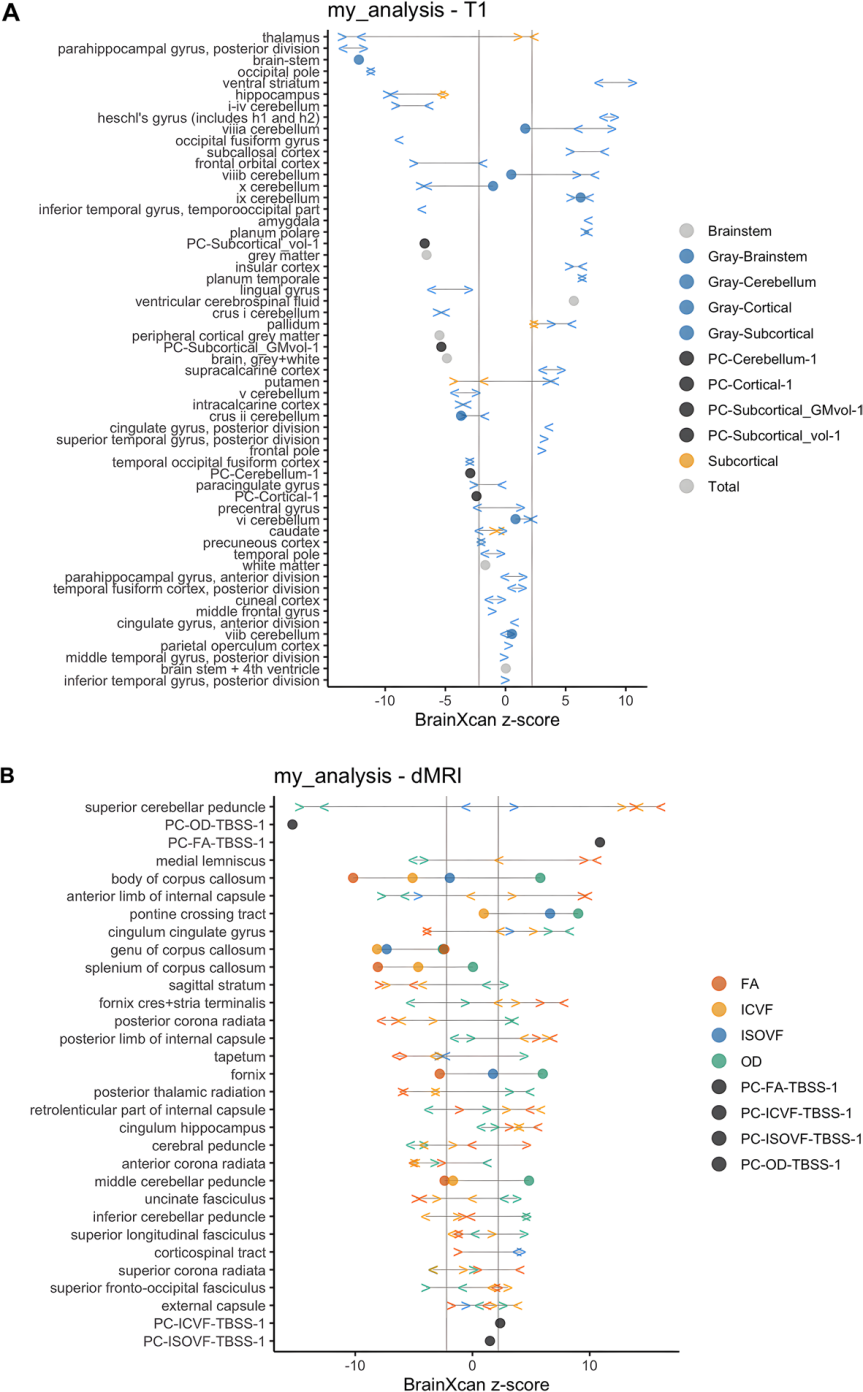
BrainXcan analysis reveals widespread brain imaging associations with cognitive abilities. (**A**) T1-weighted structural imaging associations showing BrainXcan z-scores across brain regions. Points represent different tissue categories: cortical gray matter (blue), subcortical gray matter (orange), cerebellar gray matter (green), brainstem (light blue), and principal components of structural volumes (black/gray). Significant associations span multiple brain regions including parahippocampal gyrus, thalamus, hippocampus, frontal and temporal cortices, and cerebellar regions. (**B**) Diffusion MRI (dMRI) associations displaying BrainXcan z-scores for white matter microstructure measures. Points represent different diffusion parameters: FA (fractional anisotropy, orange), ICVF (intracellular volume fraction, yellow), ISOVF (isotropic volume fraction, blue), OD (orientation dispersion, green), and tract-based spatial statistics principal components (black). Significant associations include corpus callosum, internal capsule, cerebellar peduncles, and major white matter tracts. Key findings: Among 261 brain imaging phenotypes, 187 showed significant associations (FDR < 0.05), achieving a 71.6% significance rate. Analysis covered 109 T1 structural and 152 diffusion MRI phenotypes. Results demonstrate widespread distributed neural foundations of cognitive abilities across both gray matter volumes and white matter microstructure, supporting whole-brain network theories of cognition rather than localized cognitive modules.

**Fig. 13:**
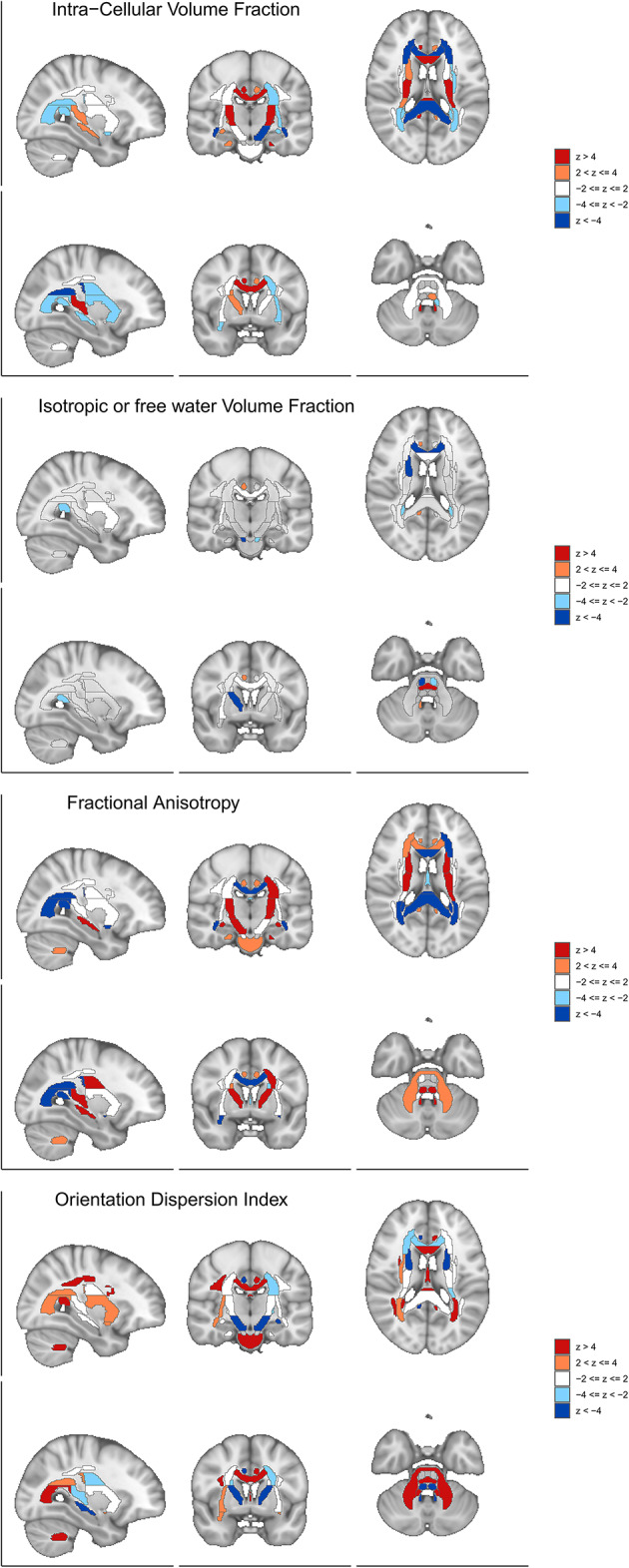
Spatial distribution of diffusion MRI associations with cognitive abilities. Brain maps showing BrainXcan z-scores for four diffusion MRI parameters across sagittal, coronal, and axial views. Red indicates positive associations (higher parameter values with increased cognitive abilities), blue indicates negative associations. (a) Intra-Cellular Volume Fraction (ICVF) showing positive associations in deep white matter regions including corpus callosum and internal capsule, indicating higher axonal density correlates with better cognition. (b) Isotropic Volume Fraction (ISOVF) demonstrating negative associations across white matter regions, indicating lower free water content associates with enhanced cognitive performance. (c) Fractional Anisotropy (FA) revealing strong positive associations in major white matter tracts including corpus callosum, internal capsule, and association fibers, confirming white matter integrity supports cognition. (d) Orientation Dispersion Index (OD) showing spatially heterogeneous associations with both positive and negative patterns across different white matter regions. Results support the connectome hypothesis that cognitive abilities depend on white matter microstructure integrity and efficient neural connectivity.

**Fig. 14:**
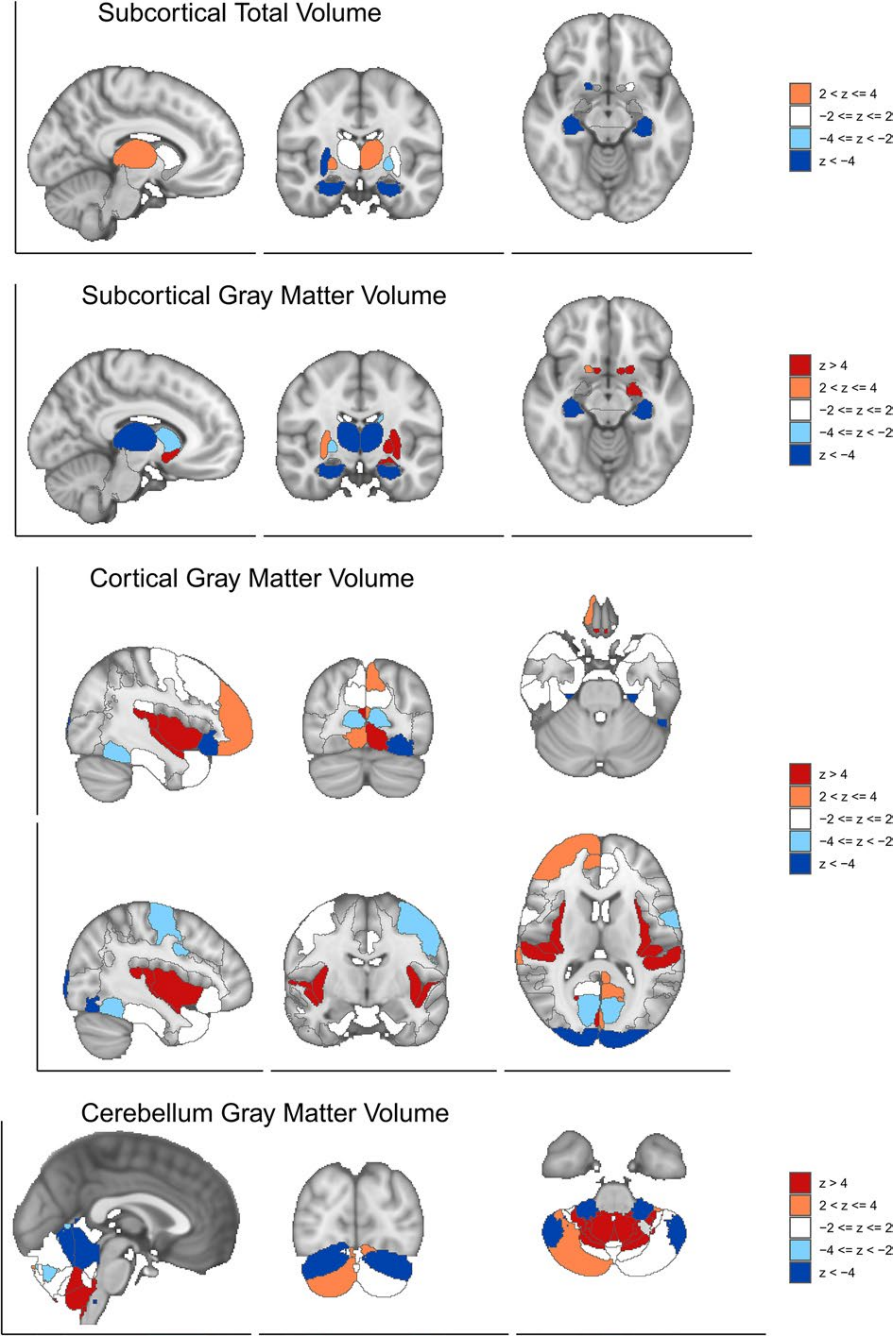
Structural brain volume associations with cognitive abilities across brain regions. Brain maps displaying BrainXcan z-scores for structural MRI volumes across four anatomical categories. Color scale represents association strength: red (z > 4, strong positive), orange (2 < z ≤ 4, moderate positive), light blue (-4 ≤ z < -2, moderate negative), and dark blue (z < -4, strong negative). (a) Subcortical Total Volume showing positive associations in hippocampus and thalamus (orange/red), with negative associations in lateral regions (blue), indicating that larger hippocampal and thalamic volumes associate with better cognitive performance. (b) Subcortical Gray Matter Volume revealing strong positive associations in hippocampus and caudate nucleus (red), and negative associations in putamen and other striatal regions (blue), suggesting differential roles of subcortical structures in cognition. (c) Cortical Gray Matter Volume demonstrating widespread associations across multiple cortical regions, with strong positive associations in frontal and temporal cortices (red) and negative associations in parietal and occipital regions (blue), reflecting distributed cortical contributions to cognitive abilities. (d) Cerebellum Gray Matter Volume showing complex positive and negative association patterns across cerebellar lobules, with positive associations in vermis and lateral hemispheres (red/orange) and negative associations in posterior regions (blue). Results reveal 48 cortical, 13 subcortical, 10 subcortical structural, and 27 cerebellar gray matter regions participating in cognitive function, supporting whole-brain distributed neural architecture rather than localized cognitive centers.

**Fig. 15:**
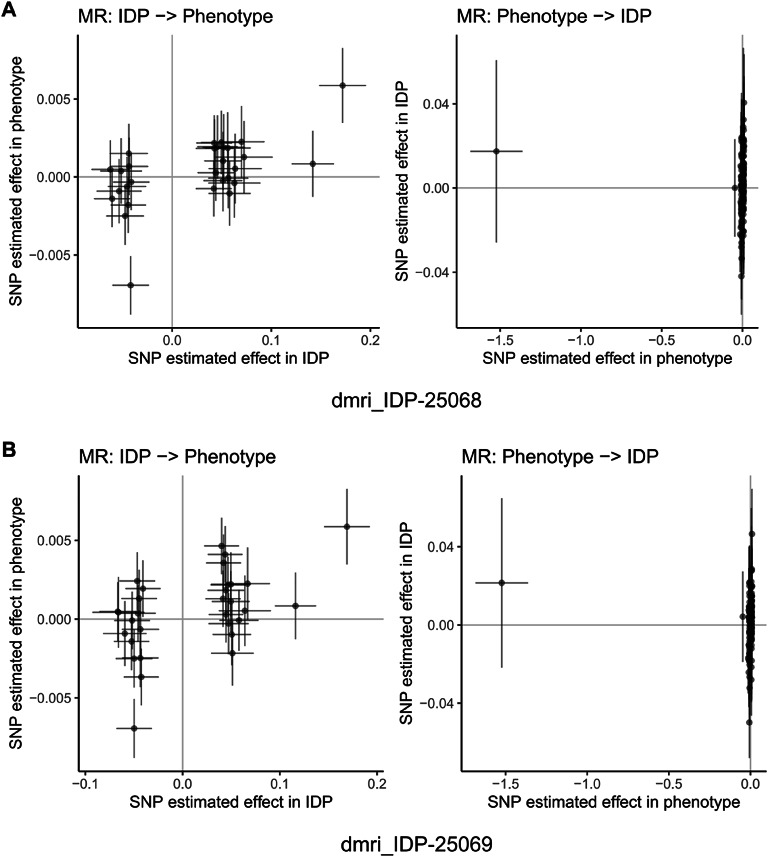

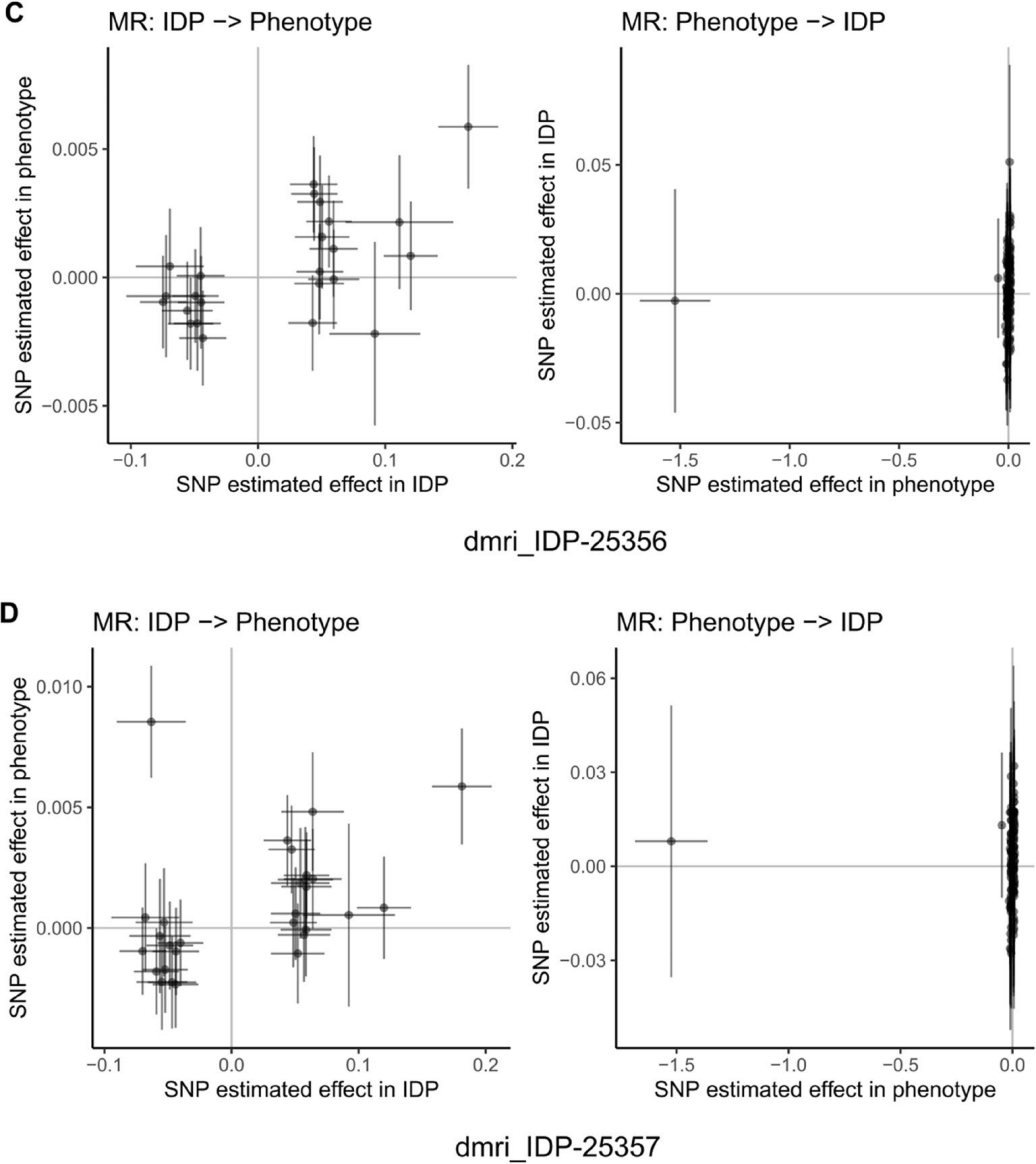

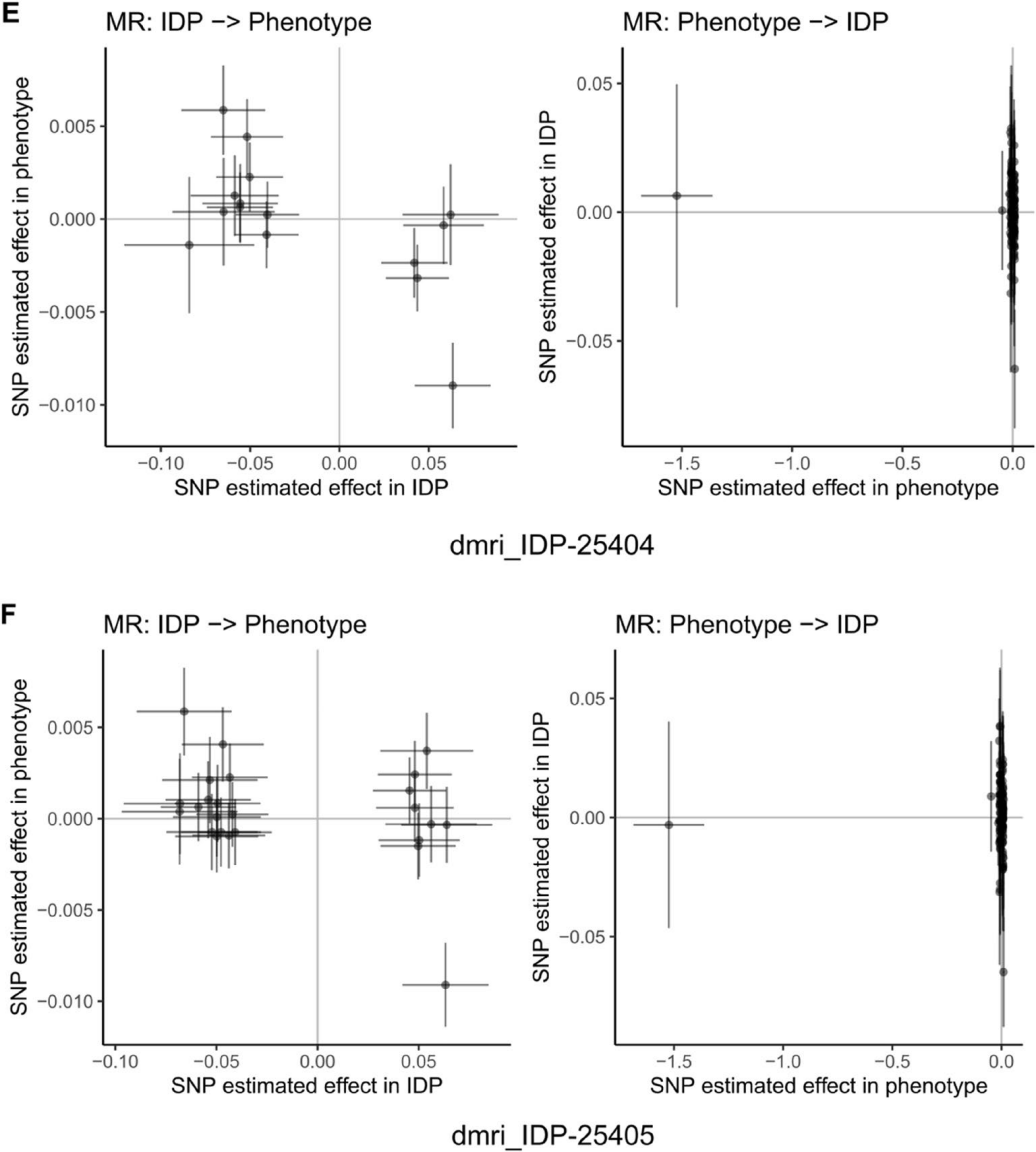

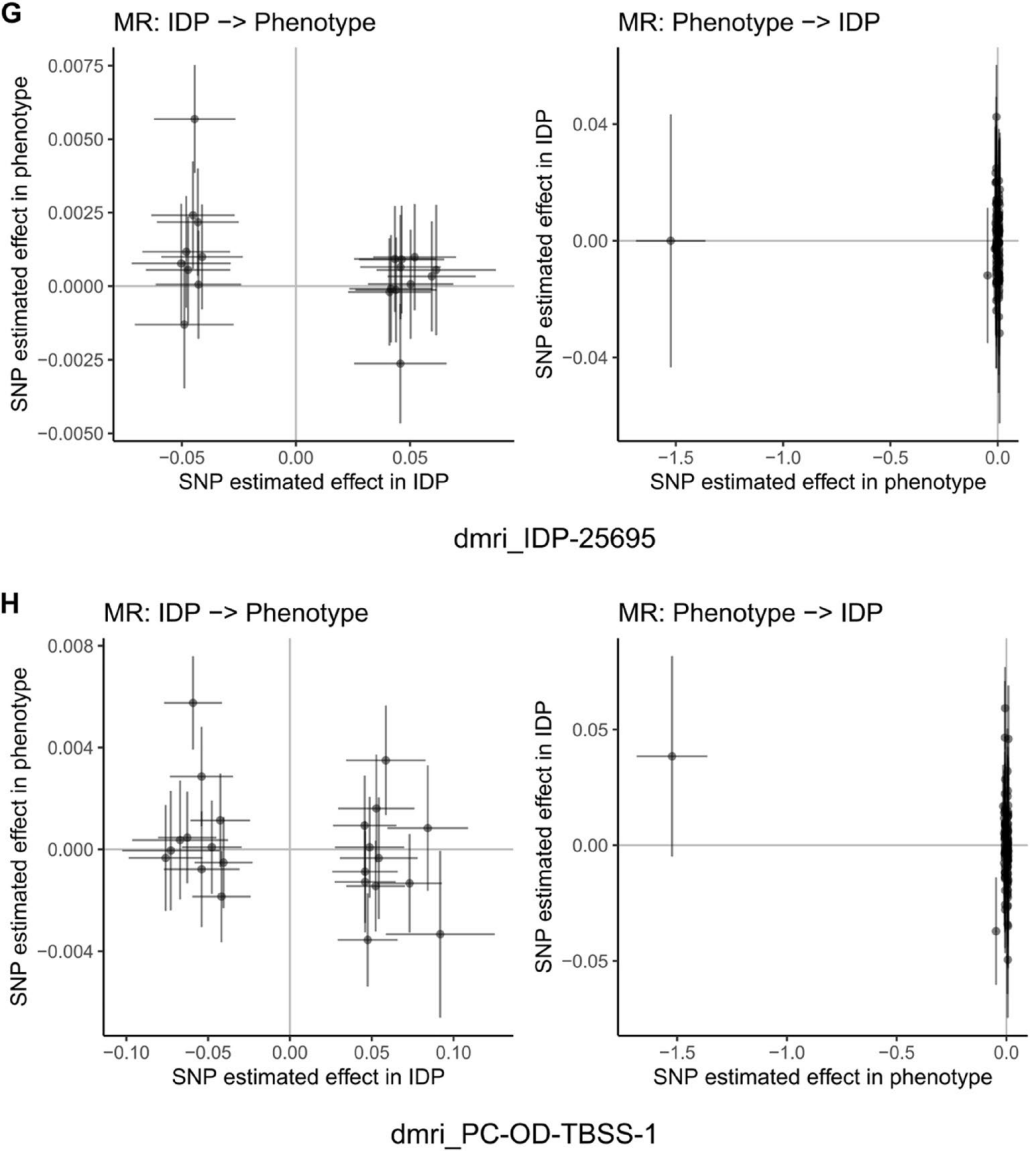

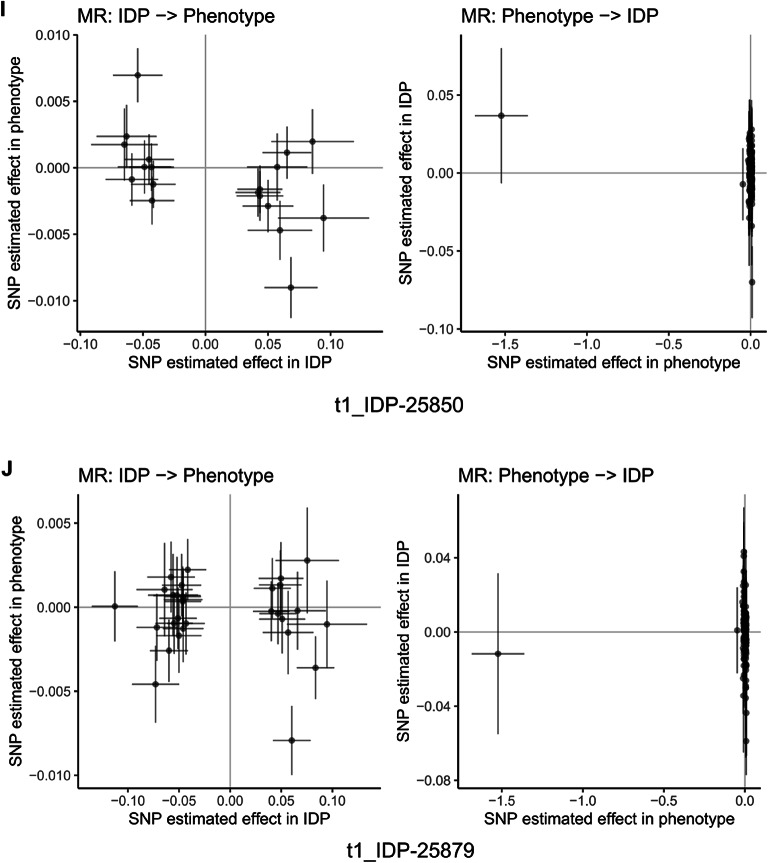
Mendelian randomization analysis of bidirectional causal relationships between cognitive abilities and brain imaging phenotypes. Scatter plots showing bidirectional Mendelian randomization (MR) analysis for representative brain imaging-derived phenotypes (IDPs) from UK Biobank. Each panel displays two analyses: left plot shows IDP as exposure and cognitive abilities as outcome (IDP → Phenotype), right plot shows reverse direction with cognitive abilities as exposure and IDP as outcome (Phenotype → IDP). Each point represents a genetic instrument (SNP) with effect estimates on x-axis (exposure) and y-axis (outcome). Lines represent MR estimates with confidence intervals. Panels (**A**–**J**) display representative IDPs including diffusion MRI white matter microstructure measures, tract-based spatial statistics principal components, and T1-weighted structural measures. Results demonstrate bidirectional causal relationships between cognitive abilities and brain structure across multiple imaging modalities. Among 3,935 brain imaging phenotypes analyzed, 31 showed significant associations using inverse variance weighted method (FDR < 0.05), with 77.4% showing protective effects. The bidirectional analysis provides evidence for both brain structure influencing cognitive performance and cognitive activities shaping brain structure, supporting activity-dependent neuroplasticity mechanisms.

Among 261 brain imaging phenotypes, BrainXcan analysis identified 187 significant associations (FDR < 0.05), with a significance rate of 71.6%, revealing the widespread distributed neural foundations of cognitive abilities. The analysis covered 109 T1-weighted structural imaging and 152 diffusion MRI phenotypes. Structural imaging revealed 48 cortical gray matter volumes, 13 subcortical gray matter volumes, 10 subcortical structural volumes, and 27 cerebellar gray matter regions involved in cognitive function. Diffusion imaging revealed the critical role of white matter microstructure, including extensive associations with 46 FA, 44 ICVF, 45 OD, and 13 ISOVF measures (Supplementary Table 17, Figs. 12, 13, 14, 15).

**Fig. 16:**
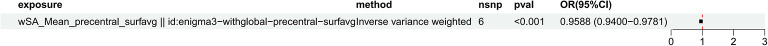
Mendelian randomization analysis of precentral gyrus surface area and cognitive abilities using ENIGMA data. Forest plot displaying Mendelian randomization results for the causal effect of precentral gyrus surface area on cognitive abilities. The analysis used weighted surface area mean of precentral gyrus (wSA_Mean_precentral_surfavg) as exposure from the ENIGMA3 consortium with global correction (enigma3-withglobal-precentral-surfavg). Method: Inverse variance weighted analysis using 6 SNPs as instrumental variables. Results: Significant protective effect with OR = 0.9588 (95% CI: 0.9400–0.9781, P = 3.33 × 10⁻^5^), indicating that genetically predicted larger precentral gyrus surface area is associated with improved cognitive performance. The confidence interval excludes 1.0, confirming statistical significance. This finding provides neuroanatomical evidence for “embodied cognition” theory, suggesting that motor cortex structure (precentral gyrus) causally influences cognitive abilities, supporting cognitive-motor co-evolution hypothesis. Based on 51,665 individuals from ENIGMA cortical morphology cohort.

ENIGMA cortical morphology cohort: Based on precentral gyrus surface area data from 51,665 individuals, we assessed its causal relationship with cognitive abilities using 5 Mendelian randomization methods. The inverse variance weighted method showed a strong protective effect (OR = 0.9588, 95% CI: 0.9400–0.9781, P = 3.33 × 10^–5^). Sensitivity analyses using weighted median (OR = 0.9612, P = 0.002), MR-Egger, and weighted mode methods demonstrated consistent directionality, with no evidence of significant directional pleiotropy (complete sensitivity analysis results in Supplementary Table 18, Fig. 16).

**Fig. 17:**
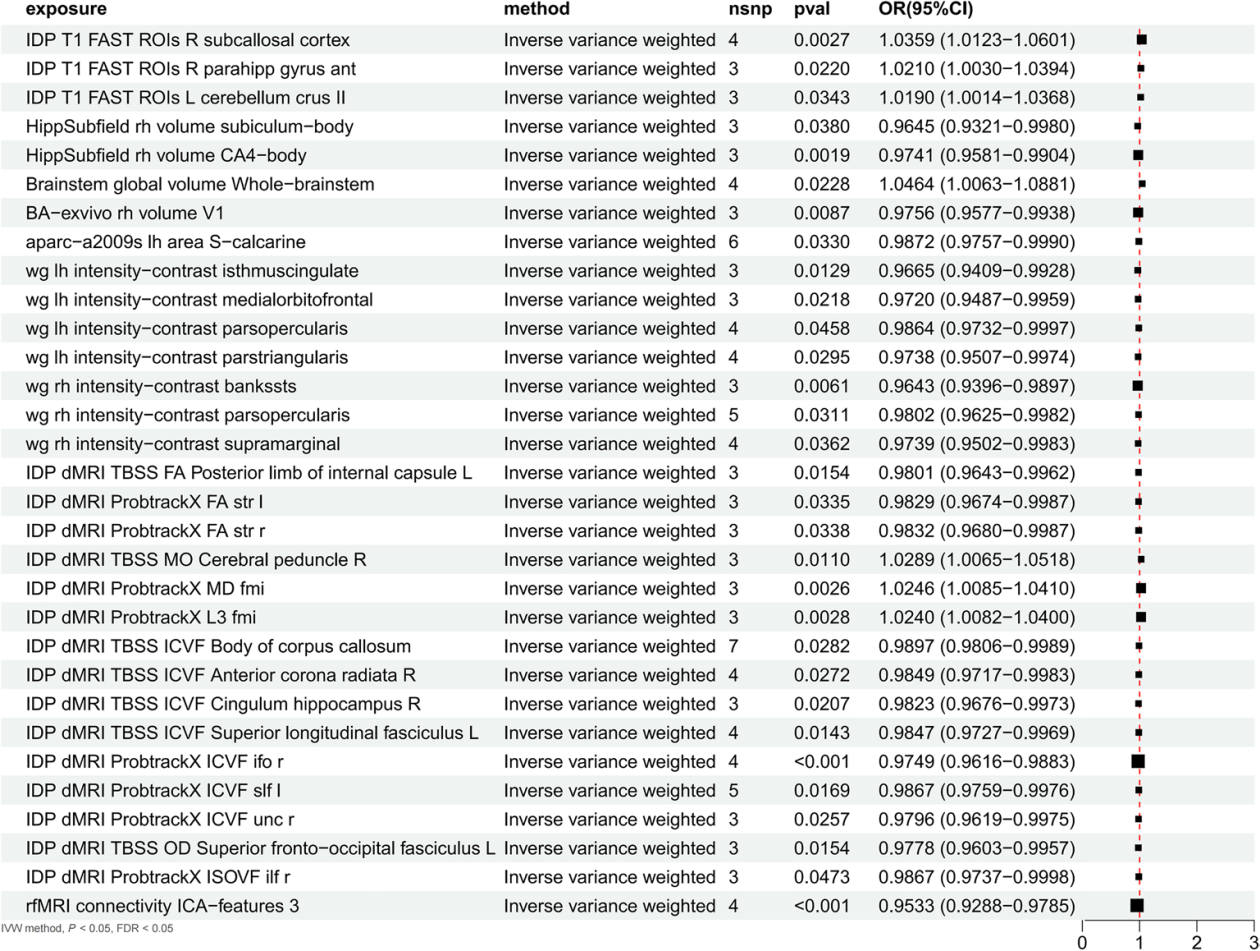
UK Biobank multimodal brain imaging associations with cognitive abilities. Forest plot displaying Mendelian randomization results for 31 brain imaging-derived phenotypes significantly associated with cognitive abilities from UK Biobank using inverse variance weighted (IVW) method (FDR < 0.05). Each row shows exposure phenotype, method, number of SNPs (nsnp), P-value, and odds ratio with 95% confidence intervals. Points represent effect estimates with confidence intervals extending horizontally; vertical dashed line at OR = 1.0 indicates no effect. Phenotype categories include: T1 structural imaging (subcallosal cortex, parahippocampal gyrus, cerebellum), hippocampal subfields (subiculum-body, CA4-body), brainstem (whole brainstem global volume), visual cortex (V1), white–gray matter contrast (multiple cortical regions including isthmus cingulate, medial orbitofrontal, pars opercularis, pars triangularis, banks superior temporal sulcus, supramarginal), diffusion MRI TBSS measures (FA, ICVF, OD in various white matter tracts), diffusion MRI tractography (multiple ProbtrackX measures), and functional connectivity (rfMRI connectivity ICA-features). Key findings: 24 phenotypes (77.4%) show protective effects (OR < 1), indicating that genetic predisposition to higher cognitive abilities associates with better brain structural and functional integrity. Notable protective associations include hippocampal subfields (OR ~ 0.97), white matter integrity measures (OR ~ 0.98), and functional connectivity (OR = 0.9533). Few phenotypes show risk associations (OR > 1), primarily in structural volumes and diffusion measures. Results support whole-brain network coordination underlying cognitive abilities across multiple imaging modalities.

UK Biobank multimodal imaging cohort: Among 3,935 brain imaging-derived phenotypes, we identified 31 phenotypes significantly associated with cognitive abilities (IVW method, FDR < 0.05). These associations encompassed T1 structural imaging (3), hippocampal subfields (2), brainstem (1), visual cortex (1), cortical parcellation (1), white–gray matter contrast (7), diffusion MRI TBSS measures (7), diffusion MRI tractography measures (8), and functional connectivity (1), with 24 phenotypes showing protective effects (77.4%). Sensitivity analyses confirmed robustness: MR-Egger intercept tests showed no evidence of directional pleiotropy (all P > 0.05), Cochran’s Q statistics indicated acceptable heterogeneity, and effect direction consistency across IVW, weighted median, and weighted mode methods was observed in the majority of associations (complete results in Supplementary Table 19, Fig. 17).

### Chromosome-level results

Our PRS-CS analysis revealed that cognitive ability-related genetic variant loci exhibited a significant hierarchical distribution pattern across different chromosomes. Weight analysis based on 455,310 effective SNPs showed marked differences in genetic contributions to cognitive phenotypes among chromosomes. Regarding chromosomal contributions, the first few large chromosomes displayed relatively high genetic contributions. Chromosomes 1 and 2 showed the highest total weight contributions, reaching magnitudes of 1.2 × 10^–1^ and 1.8 × 10^–1^, respectively, while chromosomes 3–5 contributed in the range of 6 × 10^–2^ to 1 × 10^–1^. The contribution levels of smaller chromosomes (such as chromosomes 21 and 22) were essentially proportional to their genomic sizes, indicating that genetic contributions to cognitive abilities are primarily determined by genome scale and gene density.

**Fig. 18:**
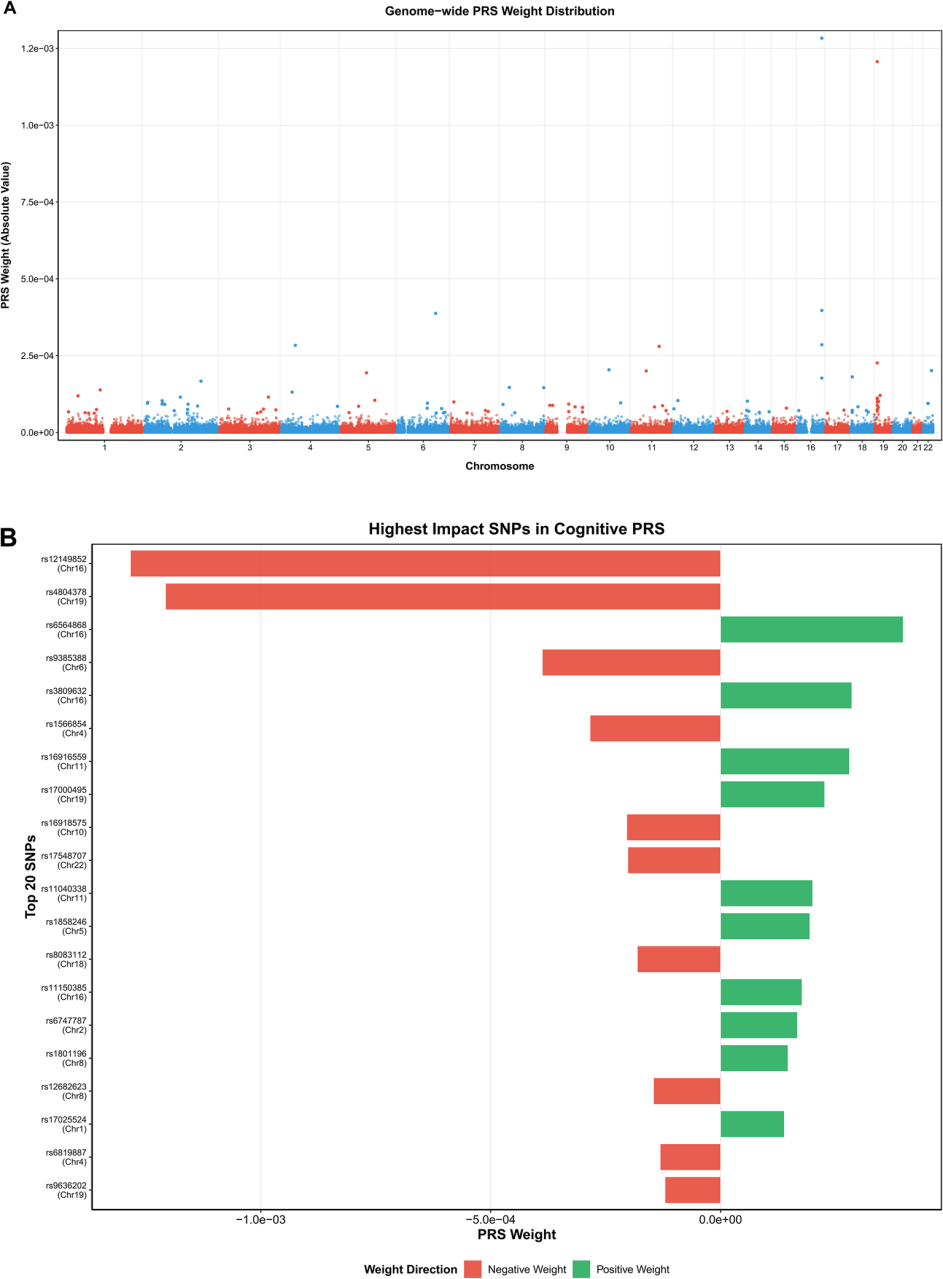

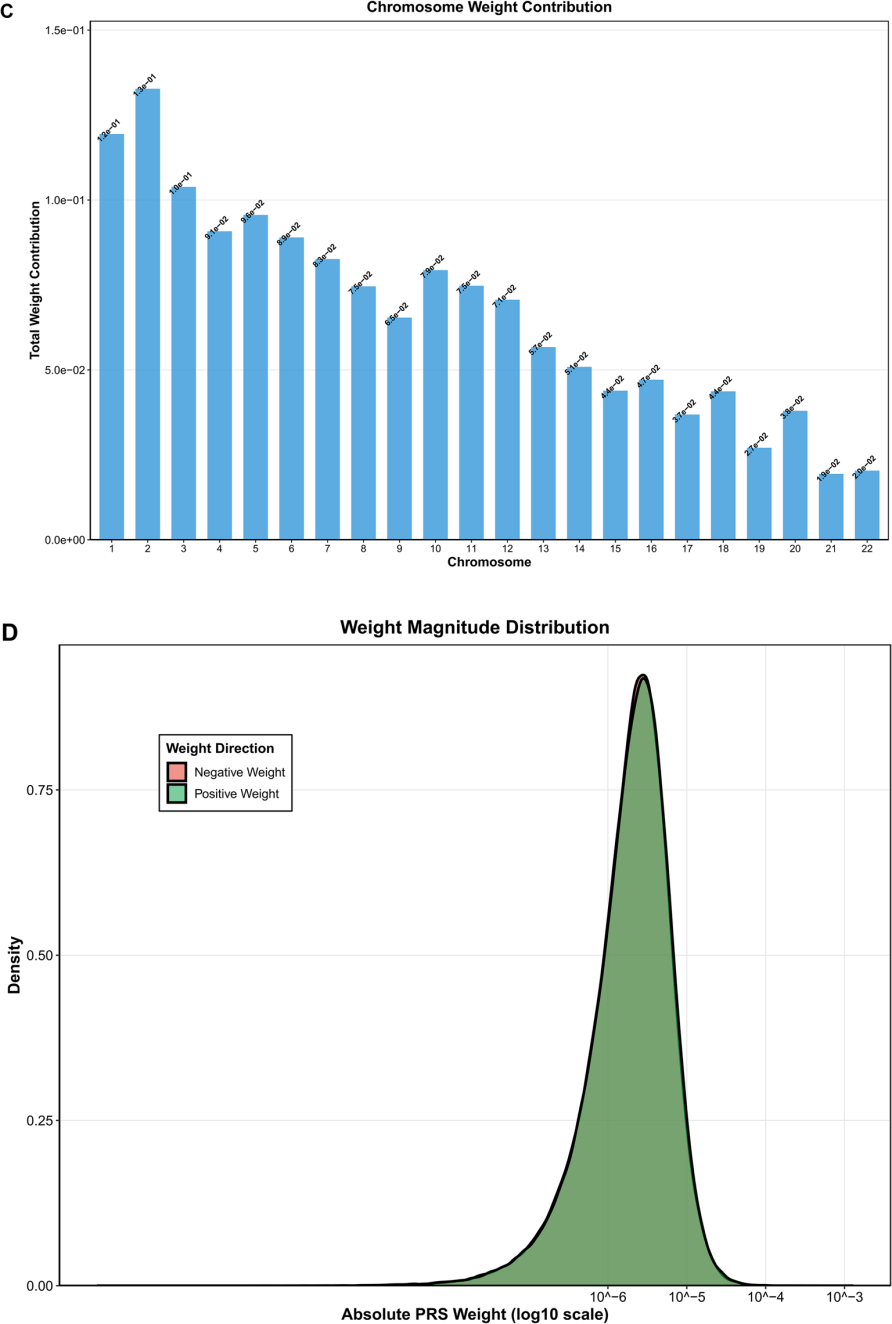
Polygenic risk score (PRS) analysis of cognitive abilities across chromosomes. (**A**) Genome-wide PRS weight distribution showing absolute weight values for 455,310 effective SNPs across chromosomes 1–22. Points are color-coded alternately (red and blue) by chromosome. The distribution demonstrates widespread polygenic architecture with variants of varying effect sizes across all chromosomes. (**B**) Highest impact SNPs in cognitive PRS displaying the top variants with largest absolute weights. Horizontal bars show negative weights (red) and positive weights (green) indicating bidirectional effects. Top 20 high-impact SNPs are primarily distributed across chromosomes 6, 9, 10, 11, 15, 16, 19, and 22. (**C**) Chromosome weight contribution showing chromosomes 1 and 2 with highest contributions (1.2 × 10⁻^1^ and 1.8 × 10⁻^1^), while chromosomes 3–5 contribute 6 × 10⁻^2^ to 1 × 10⁻^1^. (**D**) Weight magnitude distribution showing most SNPs have small effect sizes concentrated around 10⁻^5^ to 10⁻^4^.Weight direction analysis revealed 229,940 positive-weight SNPs and 225,370 negative-weight SNPs, indicating balanced bidirectional regulatory networks. Results demonstrate that cognitive genetic contributions are primarily determined by genome scale and gene density.

Weight direction analysis showed that positive-weight SNPs (229,940) and negative-weight SNPs (225,370) were relatively balanced across chromosomes, suggesting that cognitive abilities involve complex bidirectional regulatory networks. Top 20 high-impact SNPs were primarily distributed on chromosomes 6, 9, 10, 11, 15, 16, 19, 22, and other regions. These high-weight variants may exert important effects on cognitive phenotypes through influencing key neurobiological pathways (Fig. 18).

## Discussion

The genetic dissection of cognitive abilities has long been one of the core challenges in behavioral genetics and neuroscience. This study, through multivariate genomic structural equation modeling based on six cognitive-related phenotypes, identified 3,842 genome-wide significant loci, including 275 novel loci, contributing to a more comprehensive genetic map of cognitive abilities. Our single-factor model revealed that Intelligence (loading 0.992), executive function (0.879), and processing speed (0.776) constitute the core components of cognitive genetic architecture, while 13 high-confidence candidate causal variants, 33 TWAS candidate genes, 71.6% brain imaging association rate, and systematic discoveries across 857 health-related phenotypes provide multidimensional evidence for understanding the biological nature of human cognition^59^.

The latent cognitive factor identified through this approach represents shared genetic variance common to all six phenotypes, capturing pleiotropic variants that influence multiple cognitive domains through coordinated biological mechanisms. Biologically, this factor reflects fundamental neural processes—synaptic efficiency, neural connectivity, and neurotransmitter regulation—that collectively enable cognitive performance across diverse tasks. The hierarchical loading pattern, with intelligence showing highest weight, suggests the factor primarily captures efficiency of information processing and executive control. Enrichment analyses revealing convergence on neurodevelopmental pathways, synaptic organization, and transcriptional regulation indicate that genetic variation operates primarily through early brain development and synaptic function. The distributed genetic architecture across all chromosomes further supports the interpretation that cognitive abilities emerge from orchestrated functioning of multiple neural systems rather than localized brain modules. These findings contribute to our understanding of the genetic basis of cognitive abilities and provide scientific support for cognitive health management in the era of precision medicine^60^.

A major challenge in traditional cognitive genetics has been the “phenotypic heterogeneity paradox”: while psychometric evidence strongly supports the existence of general cognitive ability (g-factor)^61^, single cognitive domain GWAS have struggled to fully capture this unity. Our genomic structural equation modeling provides a potential molecular genetic approach to address this long-standing problem. The high factor loading of intelligence (0.992) provides support for Spearman’s g-factor theory, while the significant loadings of executive function (0.879) and processing speed (0.776) reveal the fine structure of cognitive genetic architecture.

This loading pattern has potential theoretical implications. It suggests that the genetic basis of human cognitive abilities may be composed of “core cognitive components”—general intelligence representing abstract reasoning ability, executive function reflecting cognitive control capacity, and processing speed embodying neural efficiency^62^. The relatively lower loadings of educational attainment (0.531), memory performance (0.670), and reaction time (0.278) do not mean they are unimportant, but rather reflect that these phenotypes contain more environmental, developmental, and measurement-specific components^63^.

Our multivariate GWAS identified 3,842 genome-wide significant loci, of which 275 (7.2%) were novel—not detected in any constituent univariate analysis. These novel loci localize predominantly to regulatory regions (68% non-coding) enriched for neurodevelopmental and synaptic pathways, representing pleiotropic variants affecting multiple cognitive domains through shared biological mechanisms. While most identified loci (92.8%) were previously reported in single-trait studies^64^, the multivariate framework enabled detection of cross-trait genetic architecture missed by single-phenotype analyses, demonstrating the value of integrative approaches for identifying coordinated genetic networks^65^.

The 3,842 genome-wide significant loci we identified were systematically annotated through FUMA software, mapping to 206 independent loci and 2,014 potential candidate genes, constructing a relatively complete discovery chain from SNPs to loci to candidate genes. Genomic control assessment provides statistical support for our findings: Mean Chi^2^ = 2.234, Lambda GC = 1.857, indicating that the observed signals are primarily driven by true polygenic heritability rather than population stratification or technical bias^66^.

The 131 “mediating loci” discovered through GWAS subtraction analysis have special theoretical value. These loci, including rs12735232 (chromosome 6), rs11210887 (chromosome 1), reached suggestive significance but did not achieve genome-wide threshold in previous educational attainment GWAS. Our analysis reveals their potential role as cognitive ability mediating loci, proposing the concept of “genetic mediation effects”: certain genetic variants may not directly influence external manifestations but exert effects indirectly through modulating internal cognitive abilities^67^.

Through dual fine-mapping strategies using SuSIE and FINEMAP, we identified 13 high-confidence candidate causal variants (average posterior probability > 0.95), providing important tools for cognitive genetics to transition from “association discovery” to “causal inference”^68^. Functional annotation of these variants reveals potential molecular regulatory networks of cognitive abilities, including neurodevelopmental regulatory axes (rs1628294 near ERRFI1, rs17374337 in TSEN2), synaptic plasticity regulatory axes (rs1566854 in CAMK2D), and transcriptional regulatory axes (rs2299297 near HOXA, chromosome 22 variant in COMT)^69^.

Our TWAS analysis identified 33 significant genes, with FOCUS fine-mapping revealing 179 candidate genes and TWAS-MAGMA intersection validation showing 67.9% overlap, establishing a relatively complete mechanism chain from genetic variants to gene expression to cognitive phenotypes^70^. Key discoveries include the epigenetic regulatory network (KANSL1 participating in histone H4K16 acetylation)^71^, neuroimmune regulatory network (TANK as TNF receptor-associated factor regulatory protein)^72^, and neurotransmitter metabolic network (SPR encoding tetrahydrobiopterin synthase)^73^.

Functional enrichment analysis revealed the “molecular ecosystem” of cognitive abilities. The 303 genes identified by MAGMA analysis showed significant enrichment in neurodevelopment-related pathways, including deletion syndrome-related gene sets and synaptic organization structure, suggesting that cognitive abilities may be an “emergent property” of the entire neurodevelopmental network^74^. Cell-type analysis showed extreme enrichment in neurons and significant enrichment in oligodendrocyte precursors, supporting “white matter plasticity” theory^75^.

LDSC partitioned heritability analysis revealed that evolutionarily conserved sequences showed extreme enrichment, indicating that cognitive-related genes are under strong selective pressure. The significant enrichment of transcriptional regulatory regions relative to coding regions reveals the “regulatory evolution” characteristics of cognitive evolution^76^.

The 71.6% significance rate (187/261) from BrainXcan analysis represents an important discovery in cognitive neuroscience theory. This result suggests that nearly three-quarters of brain imaging phenotypes are cognition-related, supporting the theoretical view that “cognition is a whole-brain network property”^77^. Diffusion imaging findings showed widespread associations across multiple parameters, suggesting that cognitive abilities may primarily reflect brain network “conduction efficiency” rather than “local function”^78^. The widespread participation of 27 cerebellar regions provides genetic support for cerebellar cognitive theory^79^.

Mendelian randomization validation in ENIGMA and UK Biobank provides important evidence for brain-cognition causal relationships. In the ENIGMA cohort, precentral gyrus surface area showed strong protective effects, providing neuroanatomical support for “embodied cognition” theory^80^. UK Biobank’s 31 significant associations across multimodal imaging, with 77.4% showing protective effects, further support the mechanism that cognitive abilities are achieved through whole-brain network coordination.

We identified 857 significant associations among 50,033 human phenotypes, constructing a comprehensive association map between cognitive abilities and human health. Cognitive abilities showed protective effects against neuropsychiatric diseases, including ADHD, providing large-scale genetic evidence for “cognitive reserve theory”. This protective association suggests that genetic variants enhancing cognitive abilities may buffer against neurodevelopmental disorder risk through improved executive control and attentional regulation, though the modest effect size indicates cognitive genetics represents only one component of complex neuropsychiatric etiology^81^. Metabolic indicator associations revealed the “cognitive-metabolic axis,” with protective associations of fasting glucose and postprandial glucose suggesting shared biological pathways linking brain energy metabolism with cognitive performance. This bidirectional relationship has potential implications for prevention strategies targeting both metabolic and cognitive health through integrated interventions^82^.

Strong protective effects of physical activity provide evidence for the “cognitive-motor co-evolution” hypothesis. This pattern may reflect genetic influences on motivation, executive function, or reward processing that jointly affect cognitive performance and health behaviors, informing precision medicine approaches that account for individual genetic predispositions^83^. From a translational perspective, these widespread phenotypic associations (857 significant) advance understanding of disease mechanisms through genetic overlap patterns. While current predictive accuracy remains insufficient for clinical risk prediction, the identified genetic pathways highlight potential targets for interventions aimed at enhancing cognitive resilience and preventing cognitive decline^84^.

Our chromosome-level PRS analysis revealed hierarchical distribution of genetic variants across chromosomes, with chromosomes 1 and 2 showing highest contributions (1.2 × 10^–1^ and 1.8 × 10^–1^), proportional to their genomic size and gene density. The balanced distribution of positive-weight (229,940) and negative-weight (225,370) SNPs across all chromosomes confirms the highly polygenic nature of cognitive abilities.

However, we acknowledge an important limitation: we did not evaluate the incremental predictive utility of chromosome-specific PRS compared to conventional genome-wide approaches in independent validation cohorts. The chromosome-level decomposition presented here serves to illustrate the distributed genomic architecture rather than to propose a superior prediction method. Future studies with independent datasets are needed to assess whether chromosome-specific risk profiles could improve individual-level prediction or identify subgroups who might benefit from targeted interventions. The genomic-wide distribution suggests that effective prediction requires integration across all chromosomes rather than focusing on specific chromosomal regions.

Despite providing new insights into cognitive ability genetics, this study has several important limitations that should be considered when interpreting the results.

Population and Generalizability Limitations: First, our sample population is primarily of European ancestry, which significantly limits the generalizability of findings to other populations and may not capture population-specific genetic variants that contribute to cognitive abilities in non-European populations. The genetic architecture of cognitive abilities may differ across populations due to different allele frequencies, linkage disequilibrium patterns, and population-specific evolutionary pressures.

Methodological and Causal Inference Limitations: Second, while we identified genetic loci associated with cognitive abilities through structural equation modeling, establishing direct causal relationships between specific genetic variants and biological mechanisms remains challenging and requires functional validation studies. Our fine-mapping analysis provides statistical evidence for potentially causal variants, but experimental validation in cellular and animal models is needed to confirm biological causality.

Genetic Coverage Limitations: Third, our analysis focuses primarily on common genetic variants (MAF > 0.01) and may miss important contributions from rare variants, structural variations, copy number variants, and other forms of genetic variation that could significantly impact cognitive abilities. Additionally, we did not account for potential effects of mitochondrial DNA variants or epigenetic modifications.

Environmental and Developmental Limitations: Fourth, although genetic factors are important, environmental factors, gene-environment interactions, and epigenetic modifications likely play substantial roles in cognitive development that were not fully addressed in this study. Factors such as educational opportunities, socioeconomic status, nutrition, and early life experiences may modify genetic effects on cognitive abilities.

Temporal and Developmental Considerations: Fifth, the cross-sectional nature of most input GWAS data limits our understanding of how genetic effects may vary across development, aging, and different life stages. Cognitive abilities are dynamic traits that change throughout the lifespan, and genetic effects may be age-dependent or show different magnitudes at different developmental periods.

Phenotypic Complexity Limitations: Sixth, our cognitive ability factor, while statistically robust, represents a simplified model of the complex, multifaceted nature of human cognition. Real-world cognitive performance involves numerous specific abilities, contextual factors, and measurement considerations that may not be fully captured by our latent factor approach.

Technical and Statistical Limitations: Finally, our multivariate approach, while innovative, relies on summary statistics from different studies with potentially varying measurement approaches, quality control procedures, and population characteristics, which could introduce heterogeneity into our analysis despite our quality control measures.

## Conclusions

This study provides evidence supporting the genetic basis of cognitive abilities through multi-level analysis. From 3,842 genetic loci to 33 candidate genes, from 71.6% brain imaging associations to 857 health-related phenotypes, we developed a cognitive biological framework spanning molecular, cellular, tissue, organ, and individual levels. These findings contribute to our understanding of human cognitive nature and provide potential foundations for evidence-based cognitive health management and human potential development in the era of precision medicine.

## Supplementary Information

Supplementary Information 1.

Supplementary Information 2.

Supplementary Information 3.

Supplementary Information 4.

Supplementary Information 5.

Supplementary Information 6.

Supplementary Information 7.

Supplementary Information 8.

Supplementary Information 9.

Supplementary Information 10.

Supplementary Information 11.

Supplementary Information 12.

Supplementary Information 13.

Supplementary Information 14.

Supplementary Information 15.

Supplementary Information 16.

Supplementary Information 17.

Supplementary Information 18.

Supplementary Information 19.

Supplementary Information 20.

Supplementary Information 21.

Supplementary Information 22.

Supplementary Information 23.

Supplementary Information 24.

Supplementary Information 25.

Supplementary Information 26.

Supplementary Information 27.

Supplementary Information 28.

Supplementary Information 29.

Supplementary Information 30.

Supplementary Information 31.

Supplementary Information 32.

Supplementary Information 33.

Supplementary Information 34.

## Data Availability

The datasets used and/or analysed during the current study are available from the corresponding author on reasonable request. The input GWAS summary statistics were obtained from publicly available sources as detailed in the Methods section, including data from the IEU OpenGWAS database and other repositories specified in the respective publications.
