# Supplementary material for: Multivariate genome-wide analysis reveals shared genetic architecture and brain structural correlates of human cognitive abilities
Source: Sci Rep. 2025 Nov 24;15:41596. doi: 10.1038/s41598-025-25509-z (PMC12644536; doi:10.1038/s41598-025-25509-z)
Supplement: Supplementary file 34 — Supplementary Information 34. [file 41598_2025_25509_MOESM34_ESM.html]

BrainXcan results for my\_analysis


# BrainXcan results for my\_analysis

# 1 BrainXcan results

In this analysis, we tested 327 structural and diffusion MRI-derived
phenotypes with cross validated spearman correlation >= 0.101.

For the main analysis we focused on 261 IDPs, which include 48
cortical volumes, 10 sub-cortical volumes, 13 sub-cortical gray matter
volumes. In below, we show the overall number of IDP by IDP subtype in
the main results.

| subtype | n |
| --- | --- |
| Brainstem | 1 |
| FA | 46 |
| Gray-Brainstem | 1 |
| Gray-Cerebellum | 27 |
| Gray-Cortical | 48 |
| Gray-Subcortical | 13 |
| ICVF | 44 |
| ISOVF | 13 |
| OD | 45 |
| PC-Cerebellum-1 | 1 |
| PC-Cortical-1 | 1 |
| PC-FA-TBSS-1 | 1 |
| PC-ICVF-TBSS-1 | 1 |
| PC-ISOVF-TBSS-1 | 1 |
| PC-OD-TBSS-1 | 1 |
| PC-Subcortical\_GMvol-1 | 1 |
| PC-Subcortical\_vol-1 | 1 |
| Subcortical | 10 |
| Total | 5 |

The p-value being used is .

Among 261 IDPs (109 T1 and 152 diffusion MRI, including the
subtype-specific PCs), 187 were significantly associated with
my\_analysis (FDR < \(0.05\)).

```
## Warning: Using `size` aesthetic for lines was deprecated in ggplot2 3.4.0.
## ℹ Please use `linewidth` instead.
## This warning is displayed once every 8 hours.
## Call `lifecycle::last_lifecycle_warnings()` to see where this warning was
## generated.
```

The top 10 associations for dMRI are

| IDP | subtype | region | side | zscore | fdr | IDP -> Phenotype | Phenotype -> IDP | link |
| --- | --- | --- | --- | --- | --- | --- | --- | --- |
| IDP-25069 | FA | superior cerebellar peduncle | left | 16.07 | 1.009e-55 | 0.003583 | 0.4643 | MR plot |
| PC-OD-TBSS-1 | PC-OD-TBSS-1 | PC-OD-TBSS-1 | NA | -15.36 | 4.209e-51 | 0.3243 | 0.1561 | MR plot |
| IDP-25404 | OD | superior cerebellar peduncle | right | -14.74 | 3.264e-47 | 0.006302 | 0.7935 | MR plot |
| IDP-25357 | ICVF | superior cerebellar peduncle | left | 14.2 | 5.886e-44 | 2.962e-08 | 0.01327 | MR plot |
| IDP-25068 | FA | superior cerebellar peduncle | right | 13.78 | 1.765e-41 | 0.001128 | 0.2165 | MR plot |
| IDP-25356 | ICVF | superior cerebellar peduncle | right | 12.76 | 9.496e-36 | 2.682e-05 | 0.05181 | MR plot |
| IDP-25405 | OD | superior cerebellar peduncle | left | -12.64 | 3.47e-35 | 0.1303 | 0.2707 | MR plot |
| PC-FA-TBSS-1 | PC-FA-TBSS-1 | PC-FA-TBSS-1 | NA | 10.87 | 2.674e-26 | NA | NA | NA |
| IDP-25065 | FA | medial lemniscus | left | 10.62 | 3.953e-25 | NA | NA | NA |
| IDP-25059 | FA | body of corpus callosum | NA | -10.18 | 3.547e-23 | NA | NA | NA |

The top 10 associations for T1 are

| IDP | subtype | region | side | zscore | fdr | IDP -> Phenotype | Phenotype -> IDP | link |
| --- | --- | --- | --- | --- | --- | --- | --- | --- |
| IDP-25879 | Gray-Subcortical | thalamus | right | -13.56 | 3.031e-40 | 0.6402 | 0.02195 | MR plot |
| IDP-25850 | Gray-Cortical | parahippocampal gyrus, posterior division | left | -13.51 | 5.286e-40 | 0.06333 | 0.1431 | MR plot |
| IDP-25892 | Gray-Brainstem | brain-stem | NA | -12.19 | 9.081e-33 | NA | NA | NA |
| IDP-25878 | Gray-Subcortical | thalamus | left | -11.97 | 1.19e-31 | NA | NA | NA |
| IDP-25851 | Gray-Cortical | parahippocampal gyrus, posterior division | right | -11.79 | 1.012e-30 | NA | NA | NA |
| IDP-25876 | Gray-Cortical | occipital pole | left | -11.21 | 6.968e-28 | NA | NA | NA |
| IDP-25877 | Gray-Cortical | occipital pole | right | -11.16 | 1.206e-27 | NA | NA | NA |
| IDP-25891 | Gray-Subcortical | ventral striatum | right | 10.58 | 5.674e-25 | NA | NA | NA |
| IDP-25887 | Gray-Subcortical | hippocampus | right | -9.798 | 1.492e-21 | NA | NA | NA |
| IDP-25886 | Gray-Subcortical | hippocampus | left | -9.286 | 1.822e-19 | NA | NA | NA |

# 2 Visualization of associations in the brain

## 2.1 T1

```
## Warning in sprintf(filenames, pages, format):
## '/var/folders/_g/xwhgcscn52x0bgnt51sslg_80000gn/T//Rtmp8lsxjrmy_analysis.vis.T1.png'里的2参数值在范围外
```

## 2.2 dMRI

```
## Warning in sprintf(filenames, pages, format):
## '/var/folders/_g/xwhgcscn52x0bgnt51sslg_80000gn/T//Rtmp8lsxjrmy_analysis.vis.dMRI.png'里的2参数值在范围外
```

# 3 BrainXcan MR Figures

## 3.1 IDP-25068

Mean FA (fractional anisotropy) in superior cerebellar peduncle
(right) on FA skeleton (from dMRI data)

```
## Warning in sprintf(filenames, pages, format):
## '/var/folders/_g/xwhgcscn52x0bgnt51sslg_80000gn/T//Rtmp8lsxjrdmri_IDP-25068.MR_vis.png'里的2参数值在范围外
```

## 3.2 IDP-25069

Mean FA (fractional anisotropy) in superior cerebellar peduncle
(left) on FA skeleton (from dMRI data)

```
## Warning in sprintf(filenames, pages, format):
## '/var/folders/_g/xwhgcscn52x0bgnt51sslg_80000gn/T//Rtmp8lsxjrdmri_IDP-25069.MR_vis.png'里的2参数值在范围外
```

## 3.3 IDP-25356

Mean ICVF (intra-cellular volume fraction) in superior cerebellar
peduncle (right) on FA (fractional anisotropy) skeleton (from dMRI
data)

```
## Warning in sprintf(filenames, pages, format):
## '/var/folders/_g/xwhgcscn52x0bgnt51sslg_80000gn/T//Rtmp8lsxjrdmri_IDP-25356.MR_vis.png'里的2参数值在范围外
```

## 3.4 IDP-25357

Mean ICVF (intra-cellular volume fraction) in superior cerebellar
peduncle (left) on FA (fractional anisotropy) skeleton (from dMRI
data)

```
## Warning in sprintf(filenames, pages, format):
## '/var/folders/_g/xwhgcscn52x0bgnt51sslg_80000gn/T//Rtmp8lsxjrdmri_IDP-25357.MR_vis.png'里的2参数值在范围外
```

## 3.5 IDP-25404

Mean OD (orientation dispersion index) in superior cerebellar
peduncle (right) on FA (fractional anisotropy) skeleton (from dMRI
data)

```
## Warning in sprintf(filenames, pages, format):
## '/var/folders/_g/xwhgcscn52x0bgnt51sslg_80000gn/T//Rtmp8lsxjrdmri_IDP-25404.MR_vis.png'里的2参数值在范围外
```

## 3.6 IDP-25405

Mean OD (orientation dispersion index) in superior cerebellar
peduncle (left) on FA (fractional anisotropy) skeleton (from dMRI
data)

```
## Warning in sprintf(filenames, pages, format):
## '/var/folders/_g/xwhgcscn52x0bgnt51sslg_80000gn/T//Rtmp8lsxjrdmri_IDP-25405.MR_vis.png'里的2参数值在范围外
```

## 3.7 PC-OD-TBSS-1

PC-OD-TBSS-1

```
## Warning in sprintf(filenames, pages, format):
## '/var/folders/_g/xwhgcscn52x0bgnt51sslg_80000gn/T//Rtmp8lsxjrdmri_PC-OD-TBSS-1.MR_vis.png'里的2参数值在范围外
```

## 3.8 IDP-25850

Volume of grey matter in Left Parahippocampal Gyrus, posterior
division

```
## Warning in sprintf(filenames, pages, format):
## '/var/folders/_g/xwhgcscn52x0bgnt51sslg_80000gn/T//Rtmp8lsxjrt1_IDP-25850.MR_vis.png'里的2参数值在范围外
```

## 3.9 IDP-25879

Volume of grey matter in Right Thalamus

```
## Warning in sprintf(filenames, pages, format):
## '/var/folders/_g/xwhgcscn52x0bgnt51sslg_80000gn/T//Rtmp8lsxjrt1_IDP-25879.MR_vis.png'里的2参数值在范围外
```
